# Supplementary material for: Weighting by Gene Tree Uncertainty Improves Accuracy of Quartet-based Species Trees
Source: Mol Biol Evol. 2022 Oct 6;39(12):msac215. doi: 10.1093/molbev/msac215 (PMC9750496; doi:10.1093/molbev/msac215)
Supplement: msac215_Supplementary_Data [file msac215_supplementary_data.pdf]

Supplementary material

Commands

Approximate Bayesian Branch Support Annotation

```
iqtree2 -s SEQ_ALIGNMENT -te GENE_TREE -m TVM+I+G4 -abayes -pre ANNOTATED_GENE_TREE
```

Note: When inferring support as a post-processing step, the same model used for inferring the tree should be used, a task that requires care when the original trees are inferred using a different tool (e.g., RAxML). TVM+I+G4 is simply an example.

Running wASTRAL

Exact commands when running on gene trees with approximate Bayesian/Bootstrap/SH-like supports.

```
astral-hybrid -x 1 -n 0.333 APPROXIMATE_BAYESIAN_ANNOTATED_GENE_TREE
astral-hybrid -x 100 -n 0 BOOTSTRAP_ANNOTATED_GENE_TREE
astral-hybrid -x 1 -n 0 SH_LIKE_ANNOTATED_GENE_TREE
```

Table S1. Counters w_*^* are defined for each node w in each gene tree, and Q is defined globally. Here, X,Y,Z are distinct colors of A, B, and C. Let u,v be the children of w; e be the parental edge of w; p be the parent of w; $\mathcal{P}_{x,w}$ be the path between x and w; $s(\mathcal{P})=1-\prod_{\hat{e}\in\mathcal{P}}(1-s(\hat{e}))$; $m(i,j)=$ MRCA of i and j. Counters for leaves are set to zero unless explicitly noted. For each counter, we show a recursive equation on top and the equivalent non-recursive definition on the bottom.

w_X	$(u_X+v_X)e^{-l(e)}$ for internal node w; $e^{-l(e)}$ for leaf node w colored X
	$\sum_i e^{-l(\mathcal{P}_{i,p})}$ for all leaf nodes i colored X under w
(w^+_{XX}, w^+_{XY})	$\left(u^+_{XX}+v^+_{XX}+u_Xv_X, u^+_{XY}+v^+_{XY}+u_Xv_Y+u_Yv_X\right)$
	$\sum_{i,j} e^{-l(\mathcal{P}_{i,j})}$ for all leaf nodes i colored X and j colored X/Y under w
(w^-_{XX}, w^-_{XY})	$\left((u^-_{XX}+v^-_{XX}+u_Xv_X)(1-s(e)), (u^-_{XY}+v^-_{XY}+u_Xv_Y+u_Yv_X)(1-s(e))\right)$
	$\sum_{i,j} e^{-l(\mathcal{P}_{i,j})}\left(1-s(\mathcal{P}_{m(i,j),p})\right)$ for all leaf nodes i colored X and j colored X/Y under w
$(w_{XX\mid Y}, w_{XY\mid Z})$	$\left((u_{XX\mid Y}+v_{XX\mid Y}+(u^+_{XX}-u^-_{XX})v_Y+u_Y(v^+_{XX}-v^-_{XX}))e^{-l(e)},\ (u_{XY\mid Z}+v_{XY\mid Z}+(u^+_{XY}-u^-_{XY})v_Z+u_Z(v^+_{XY}-v^-_{XY}))e^{-l(e)}\right)$
	$\sum_{i,j,k} e^{-l(\mathcal{P}_{i,j})-l(\mathcal{P}_{k,p})} s(\mathcal{P}_{m(i,j),m(i,k)})$ for leaf nodes i colored X, j colored X/Y, k colored Z under w, and $m(i,j)$ under $m(i,k)$
$w_{XX\mid YZ}$	$v_Xu_{YZ\mid X}+u_Xv_{YZ\mid X}+u_{XX\mid Z}v_Y+v_{XX\mid Z}u_Y+u_{XX\mid Y}v_Z+v_{XX\mid Y}u_Z +(u^+_{YZ}v^+_{XX}-u^-_{YZ}v^-_{XX})+(u^+_{XX}v^+_{YZ}-u^-_{XX}v^-_{YZ})$
	$\sum_{h,i,j,k} w_G(hi\mid jk)$ for all leaf nodes h,i colored X, j colored Y, k colored Z, and $w=$ MRCA h,i,j,k
Q	$\sum_{G\in\mathcal{G}}\sum_w (w_{AA\mid BC}+w_{BB\mid AC}+w_{CC\mid AB})$ for internal nodes w in G
	$\sum_{G\in\mathcal{G}}\sum_{h,i,j,k} w_G(hi\mid jk)$ for leaf nodes h,i,j,k in G where h,i have the same color and i,j,k have different colors; when species coloring matches all gene trees, $Q=W[A\mid B\mid C]=\sum_{G\in\mathcal{G}} W(A\mid B\mid C,G)$ (Proposition 5).

Table S2. Running time of species tree inference methods on biological datasets. We use 5.17.3 version of ASTRAL-III if not otherwise clarified.

Dataset	n	k	Method	#Cores	Wall-clock time	CPU time
OneKP	1178	410	wASTRAL-h	16	17.1 min	4.57 hr
			ASTRAL-III (5.0.3)	1	17.2 hr	17.2 hr
Canis	48	449450	wASTRAL-h	1	17.7 hr	17.7 hr
Avian	48	14446	wASTRAL-h	16	1.76 min	28.1 min
			ASTRAL-III	16	20.9 min	5.57 hr
Cetacean	98	3191	wASTRAL-h	16	35.2 sec	9.39 min
			ASTRAL-III	16	1.97 min	31.5 min
Nomiinae	32	853	wASTRAL-h	1	5.93 sec	5.93 sec
			ASTRAL-III	1	8.64 sec	8.64 sec
Lepidoptera	203	1930	wASTRAL-h	16	2.02 min	32.3 min
			ASTRAL-III	16	9.14 min	2.44 hr
Papilionidae	61	6405	wASTRAL-h	16	24.8 sec	6.61 min
			ASTRAL-III	16	1.11 min	17.8 min

Supplementary Figures and Tables

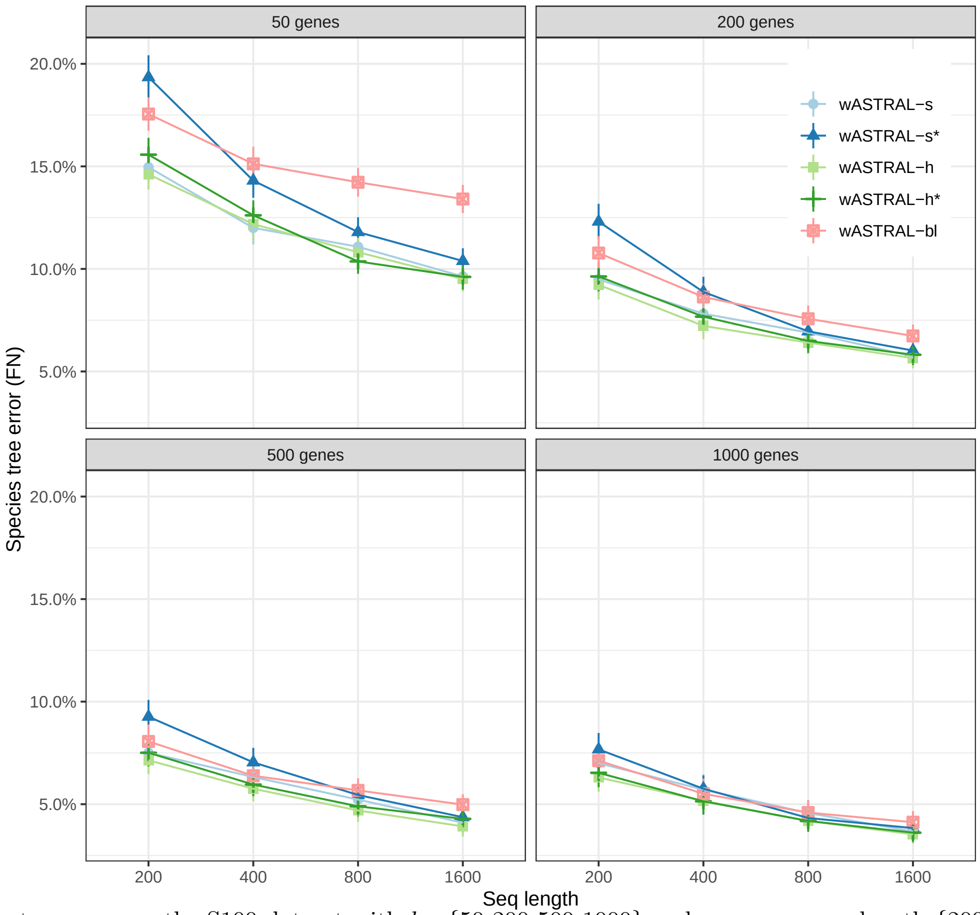

FIG. S1. Species tree error on the S100 dataset with $k = \{50, 200, 500, 1000\}$ and gene sequence length $\{200, 400, 800, 1600\}$. Top: comparison of by weighting scheme. Results with aBayes supports are labeled wASTRAL-s and wASTRAL-h; results with bootstrap support are labelled wASTRAL-s* and wASTRAL-h*. Bottom: comparison with other methods, similar to Figure 2a, but with a y-axis kept fixed.

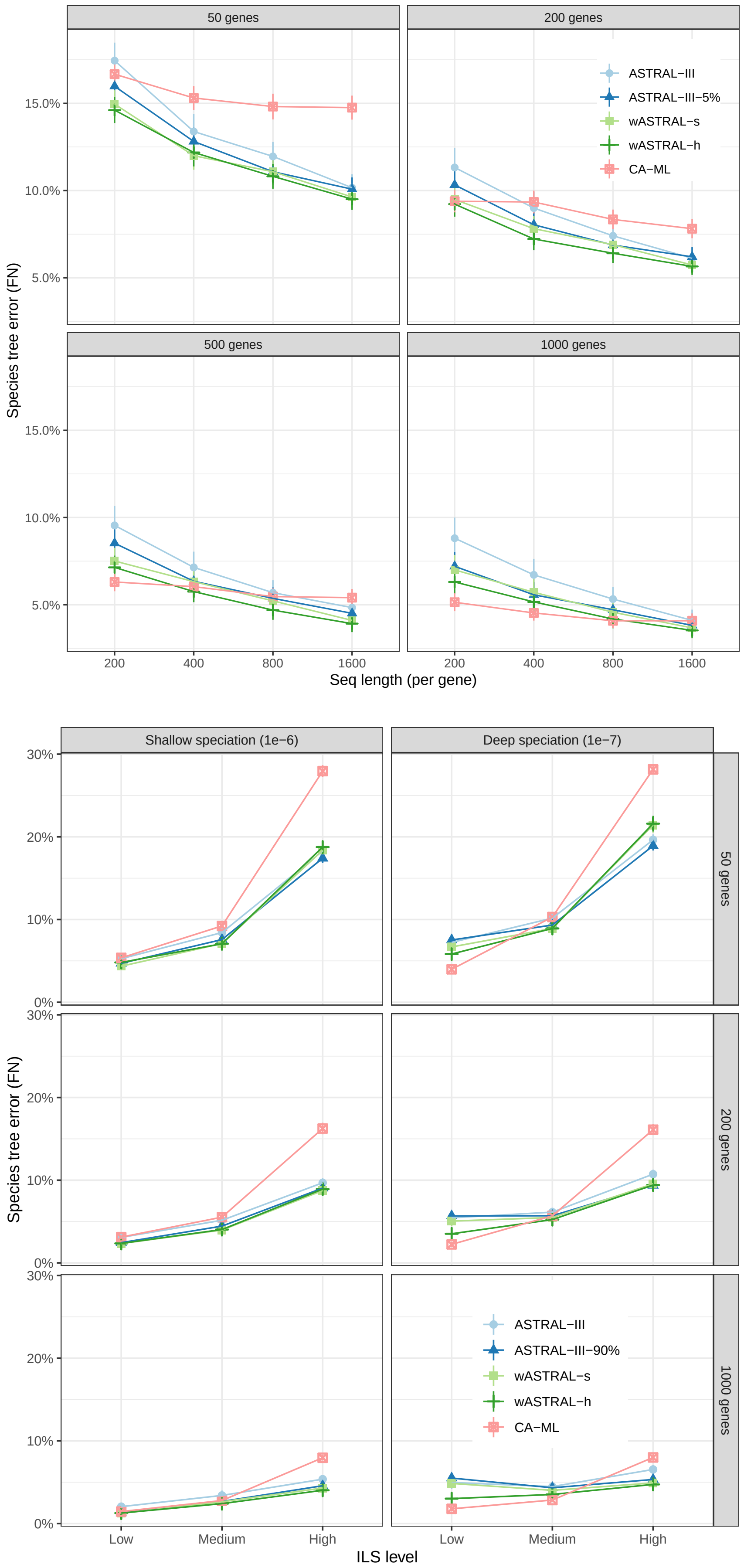

FIG. S2. Species tree error on the S100 dataset (top) and S200 dataset (bottom), similar to Figures 2 and 3 of the main paper, but with y axis kept fixed.

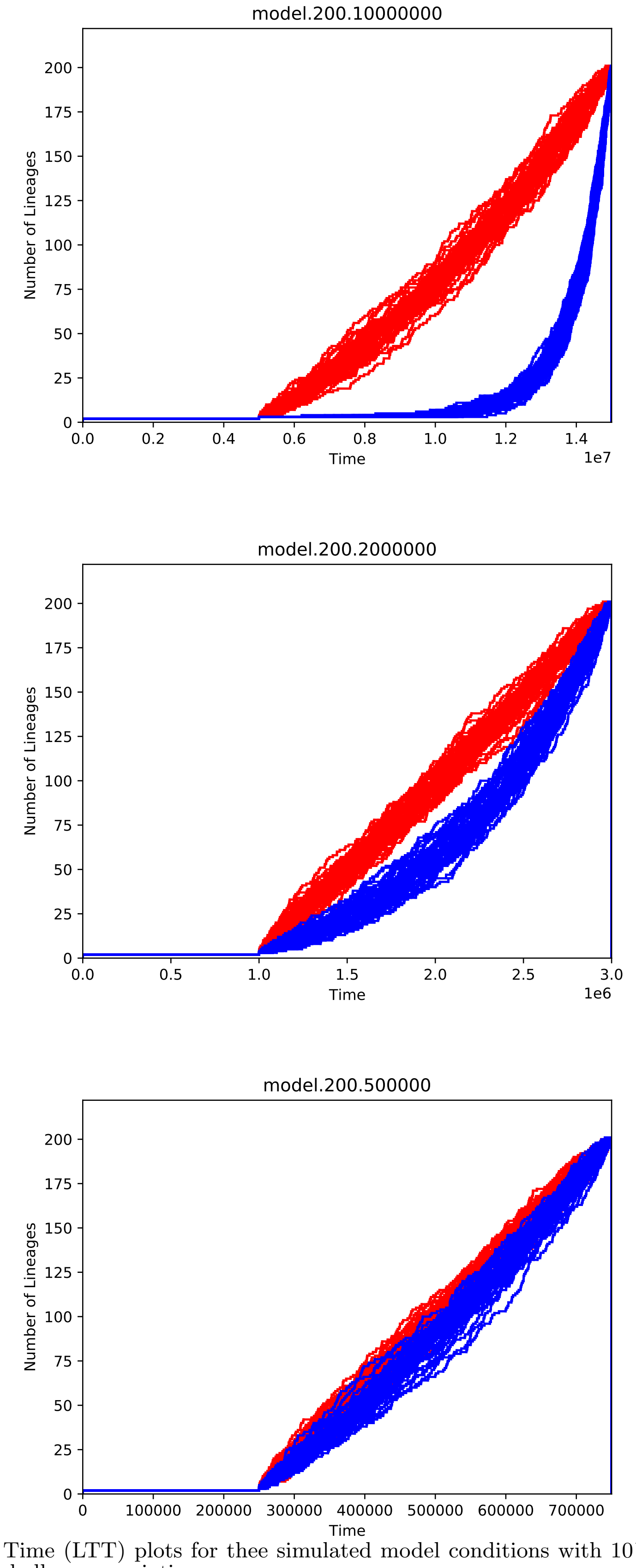

FIG. S3. Lineage Through Time (LTT) plots for thee simulated model conditions with 10^{-7} (red) and 10^{-6} (blue) rates tend to lead to deeper and shallower speciation.

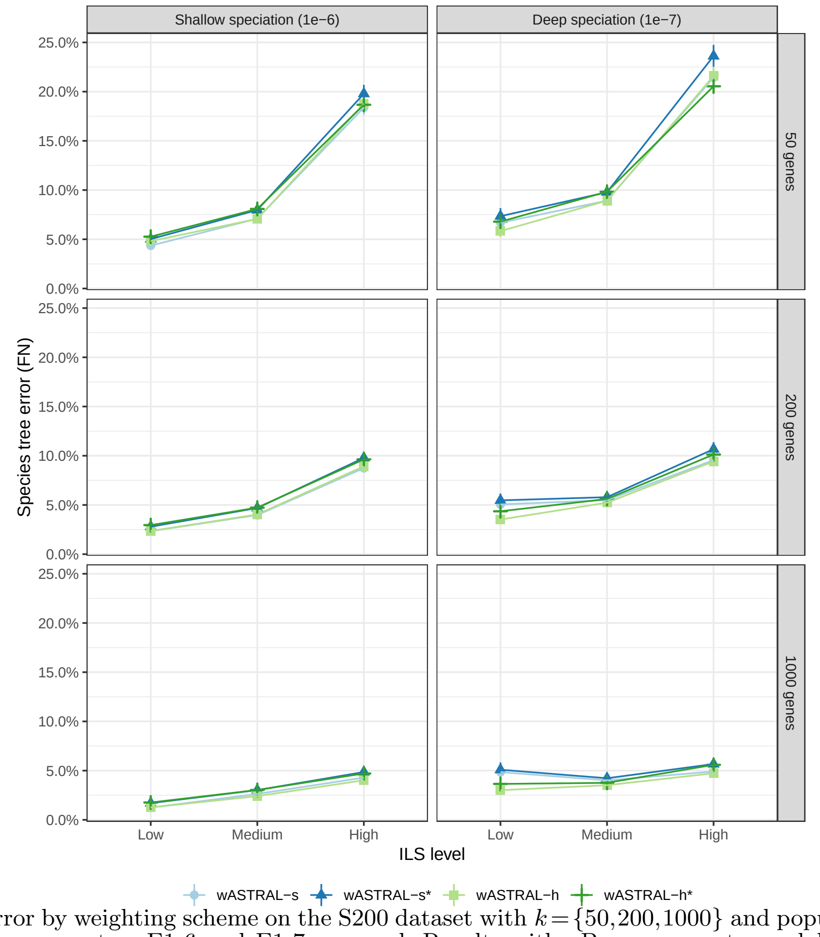

FIG. S4. Species tree error by weighting scheme on the S200 dataset with $k = \{50, 200, 1000\}$ and population size (ILS levels). Species tree shape with parameters E1-6 and E1-7 are used. Results with aBayes supports are labeled wASTRAL-s and wASTRAL-h; results with SH-like support are labeled wASTRAL-s* and wASTRAL-h*.

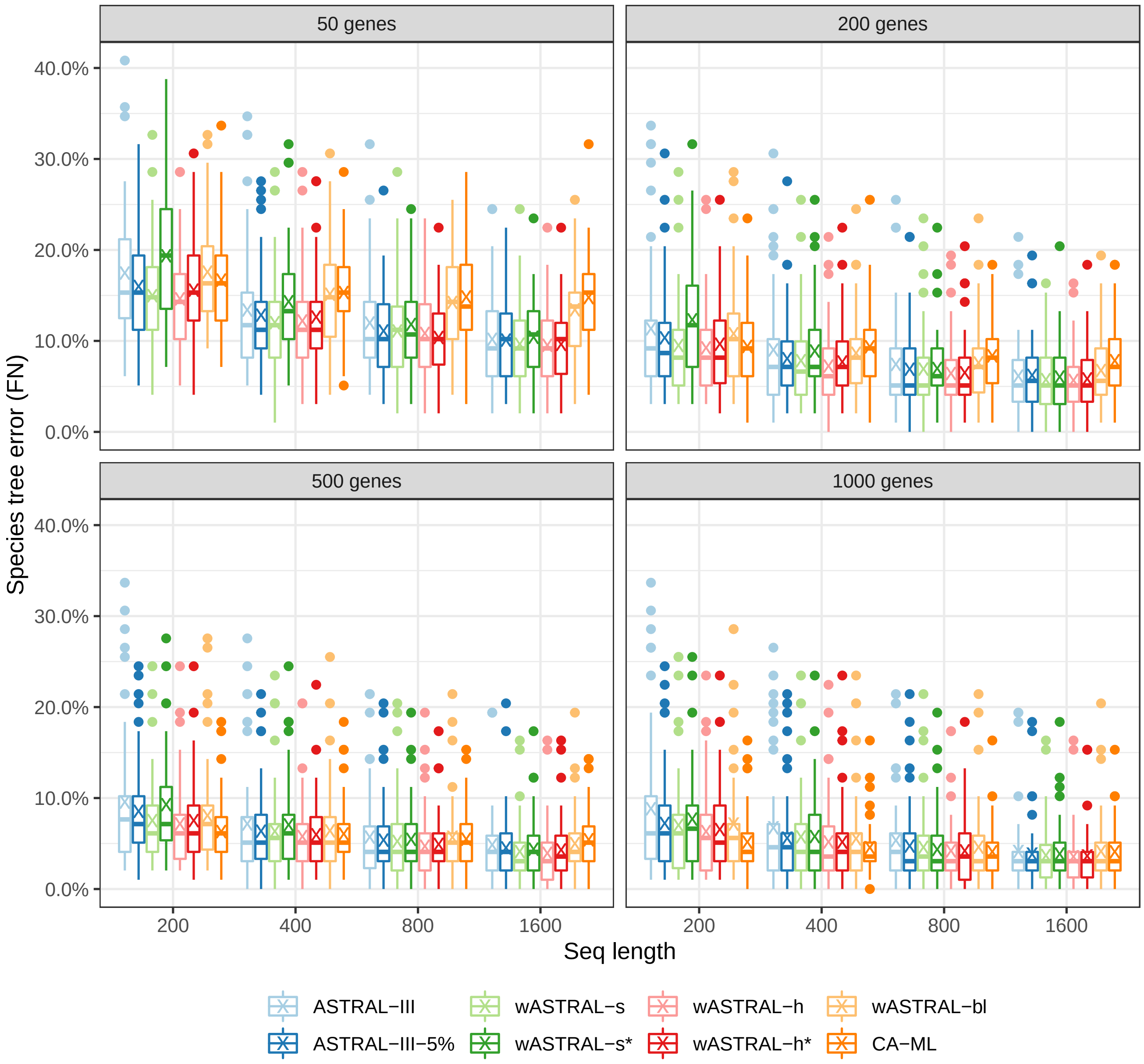

FIG. S5. Species tree error on the S100 dataset with $k=\{50,200,500,1000\}$ and gene sequence length $\{200,400,800,1600\}$. Results with aBayes supports are labelled wASTRAL-s and wASTRAL-h; results with bootstrap support are labelled wASTRAL-s* and wASTRAL-h*.

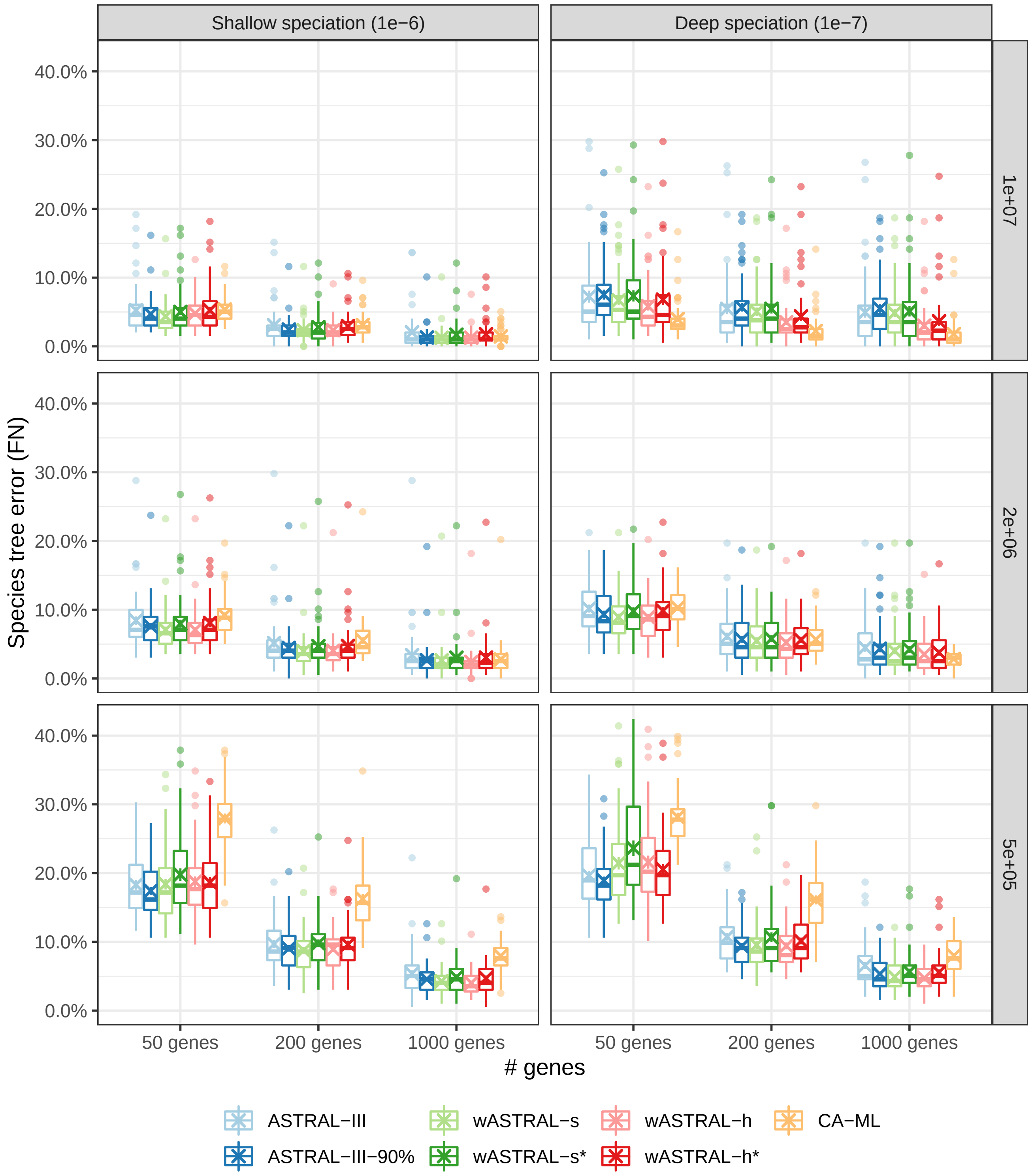

FIG. S6. Species tree error on the S200 dataset with $k = \{50, 200, 1000\}$ and population size (ILS levels). Species tree shape with parameter E1-6 and E1-7 (box columns) and ILS levels (box rows) low (1e+07), medium (2e+06), and high (5e+05) are used. Results with Bayesian supports are labeled wASTRAL-s and wASTRAL-h; results with SH-like support are labeled wASTRAL-s* and wASTRAL-h*.

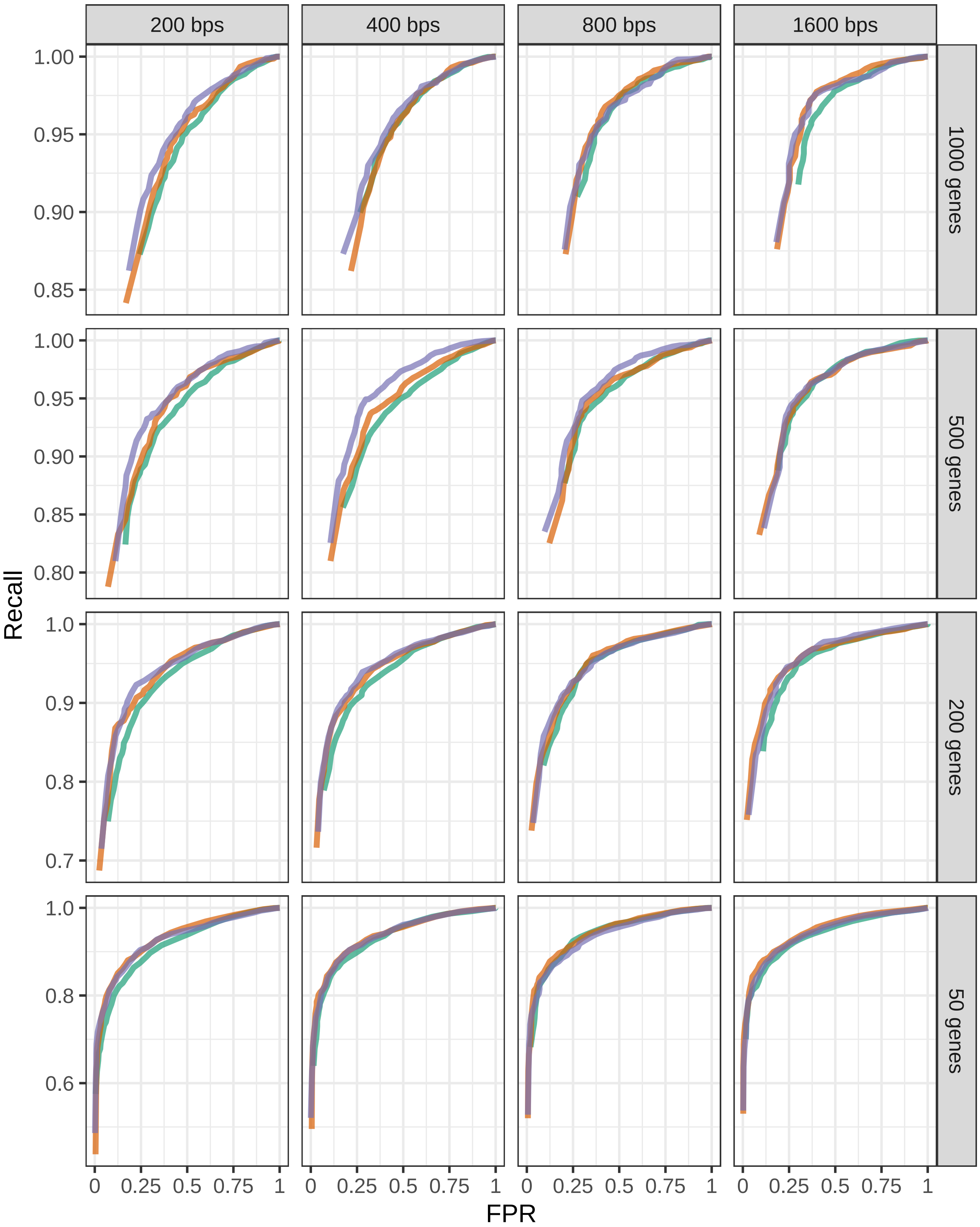

FIG. S7. ROC of S100 dataset with $k = \{50, 200, 500, 1000\}$ and gene sequence length $\{200, 400, 800, 1600\}$ as we change the threshold of support considered. Results with aBayes supports are labelled wASTRAL-s and wASTRAL-h; results with FastTree-2 bootstrap support are labelled wASTRAL-s* and wASTRAL-h*.

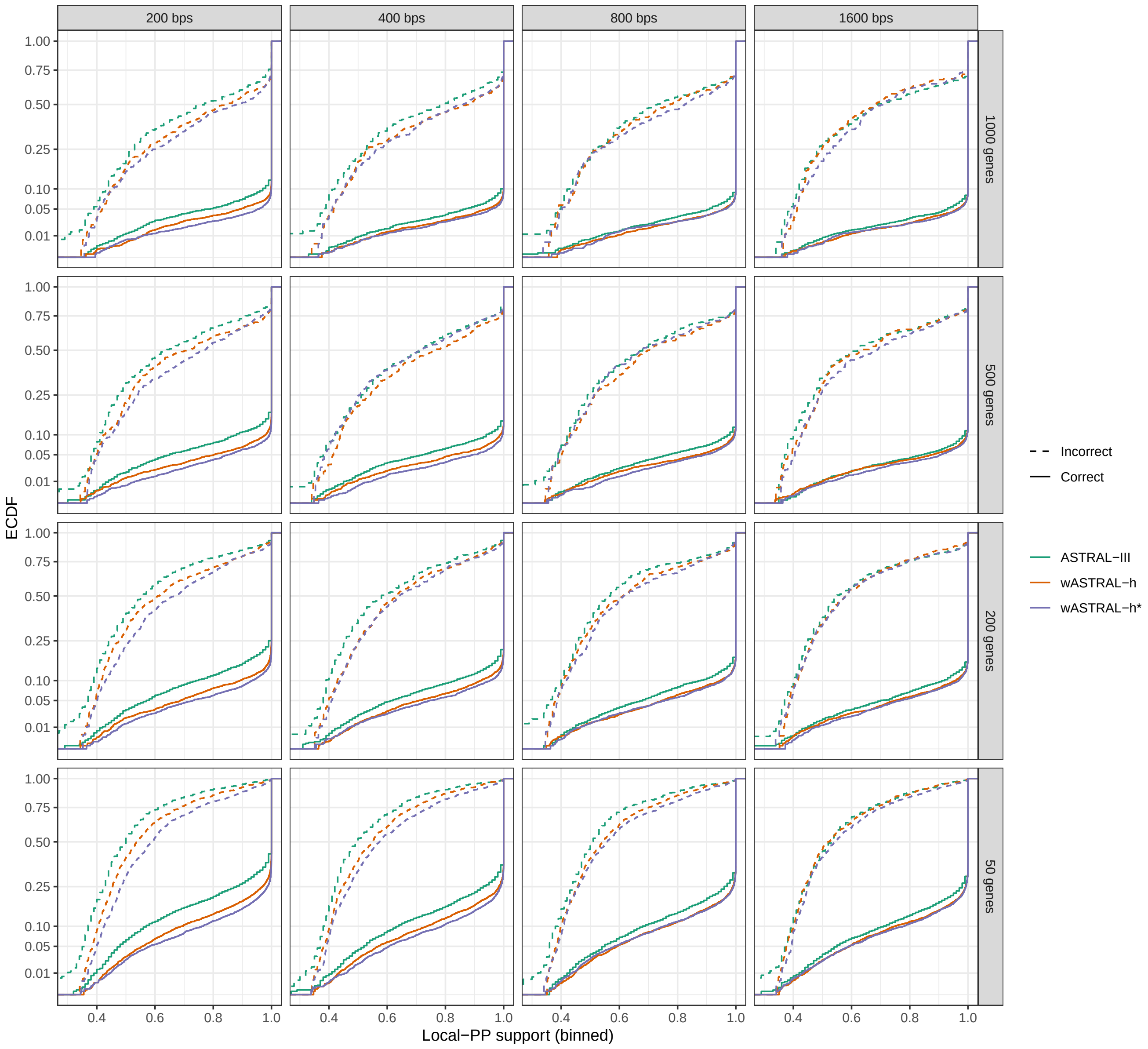

FIG. S8. ECDF of S100 dataset with $k = \{50, 200, 500, 1000\}$ and gene sequence length $\{200, 400, 800, 1600\}$. Results with aBayes supports are labelled wASTRAL-s and wASTRAL-h; results with FastTree-2 bootstrap support are labelled wASTRAL-s* and wASTRAL-h*.

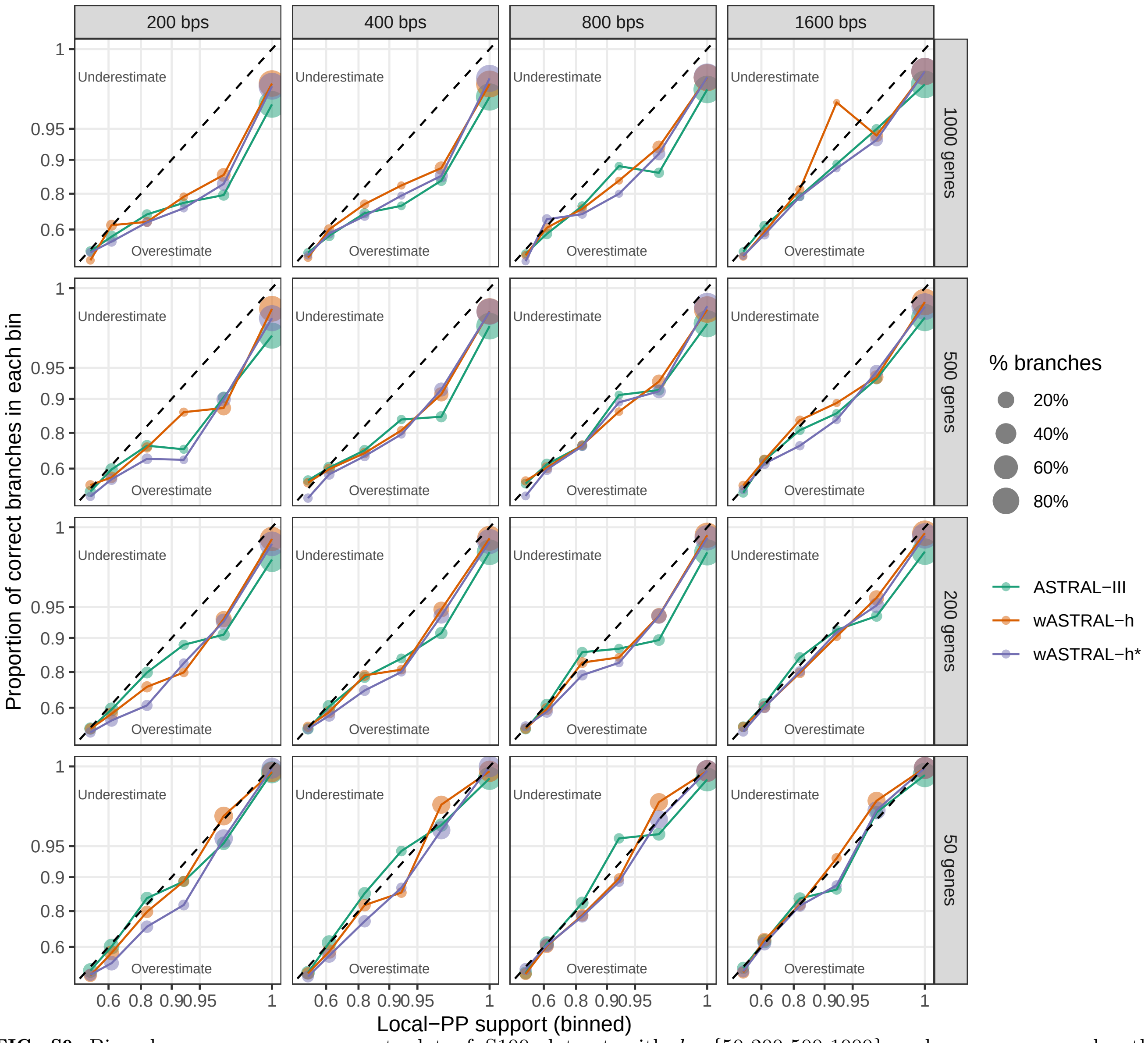

FIG. S9. Binned accuracy-verses-support plot of S100 dataset with $k=\{50,200,500,1000\}$ and gene sequence length $\{200,400,800,1600\}$. Results with aBayes supports are labelled wASTRAL-s and wASTRAL-h; results with FastTree-2 bootstrap support are labelled wASTRAL-s* and wASTRAL-h*.

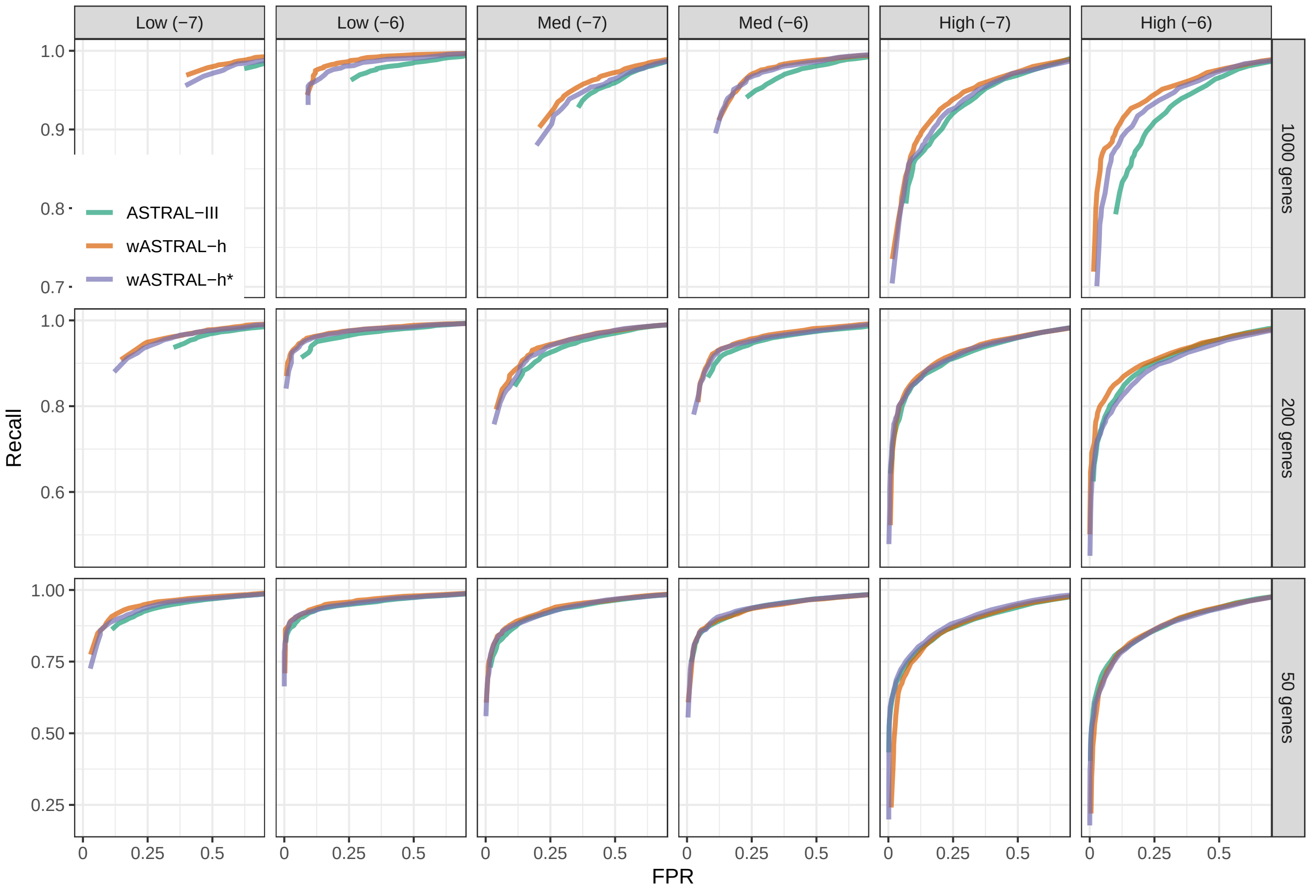

FIG. S10. ROC of S200 dataset with $k=\{50,200,1000\}$ and population size (ILS levels). Species tree shape with parameter E1-6 and E1-7 are used. Results with aBayes supports are labeled wASTRAL-h; results with SH-like support are labelled wASTRAL-h*.

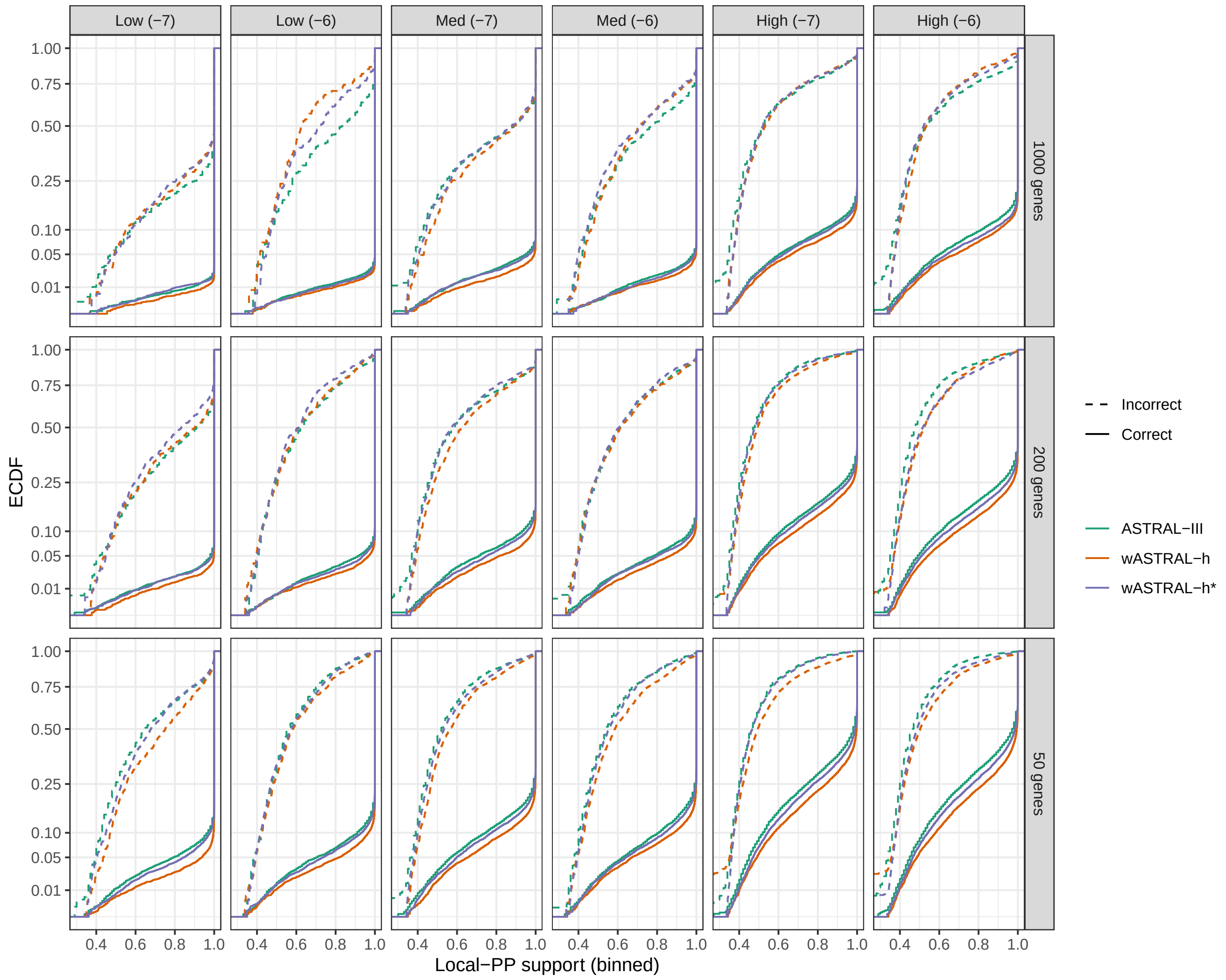

FIG. S11. ECDF of S200 dataset with $k = \{50, 200, 1000\}$ and population size (ILS levels). Species tree shape with parameter E1-6 and E1-7 (box columns) and ILS levels (box rows) low (1e+07), medium (2e+06), and high (5e+05) are used. Results with aBayes supports are labelled wASTRAL-h; results with SH-like support are labelled wASTRAL-h*.

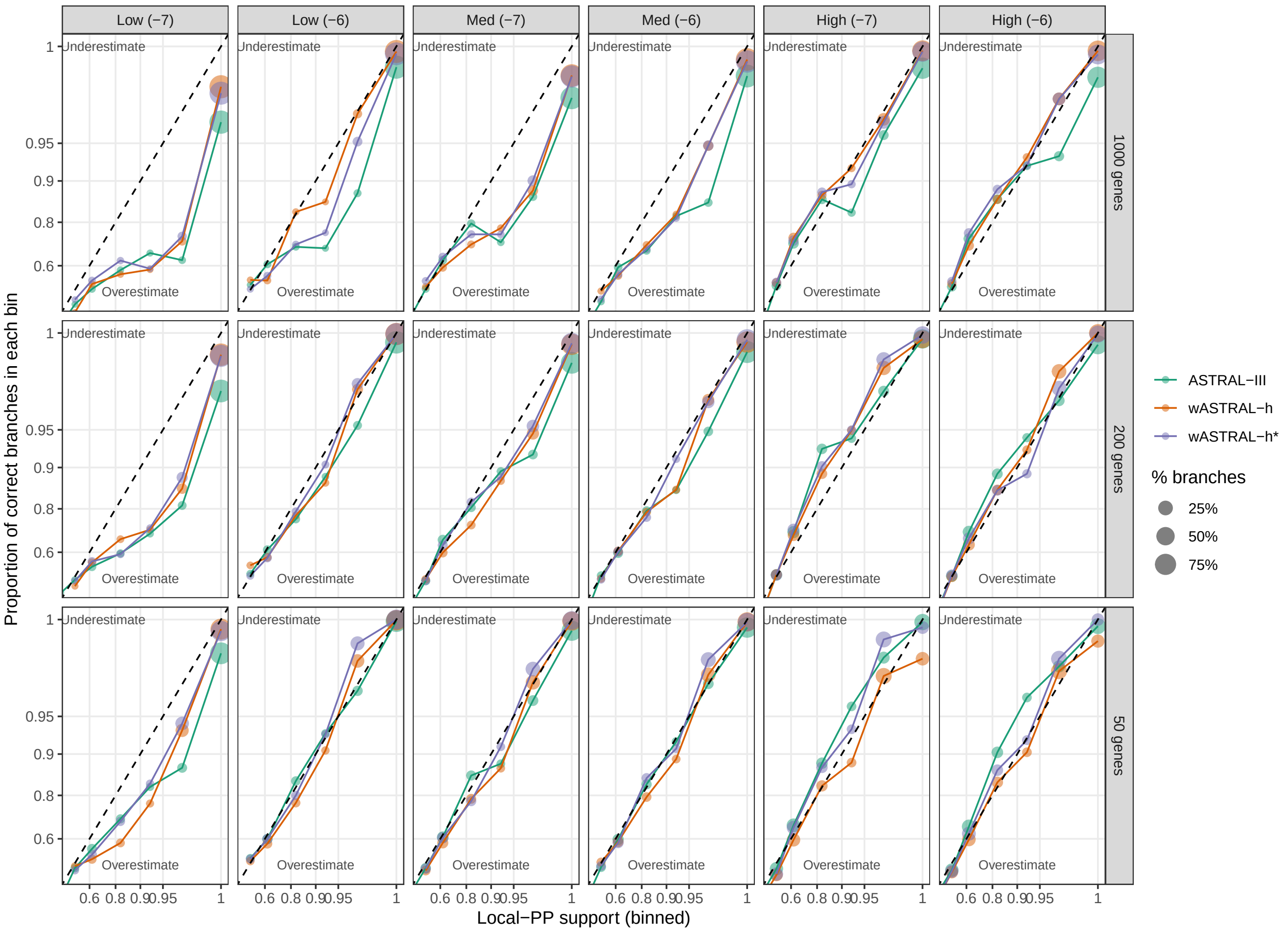

FIG. S12. Binned accuracy-verses-support plot of S200 dataset with $k = \{50, 200, 1000\}$ and population size (ILS levels). Species tree shape with parameter E1-6 and E1-7 are used. Results with aBayes supports are labeled wASTRAL-h; results with SH-like support are labeled wASTRAL-h*.

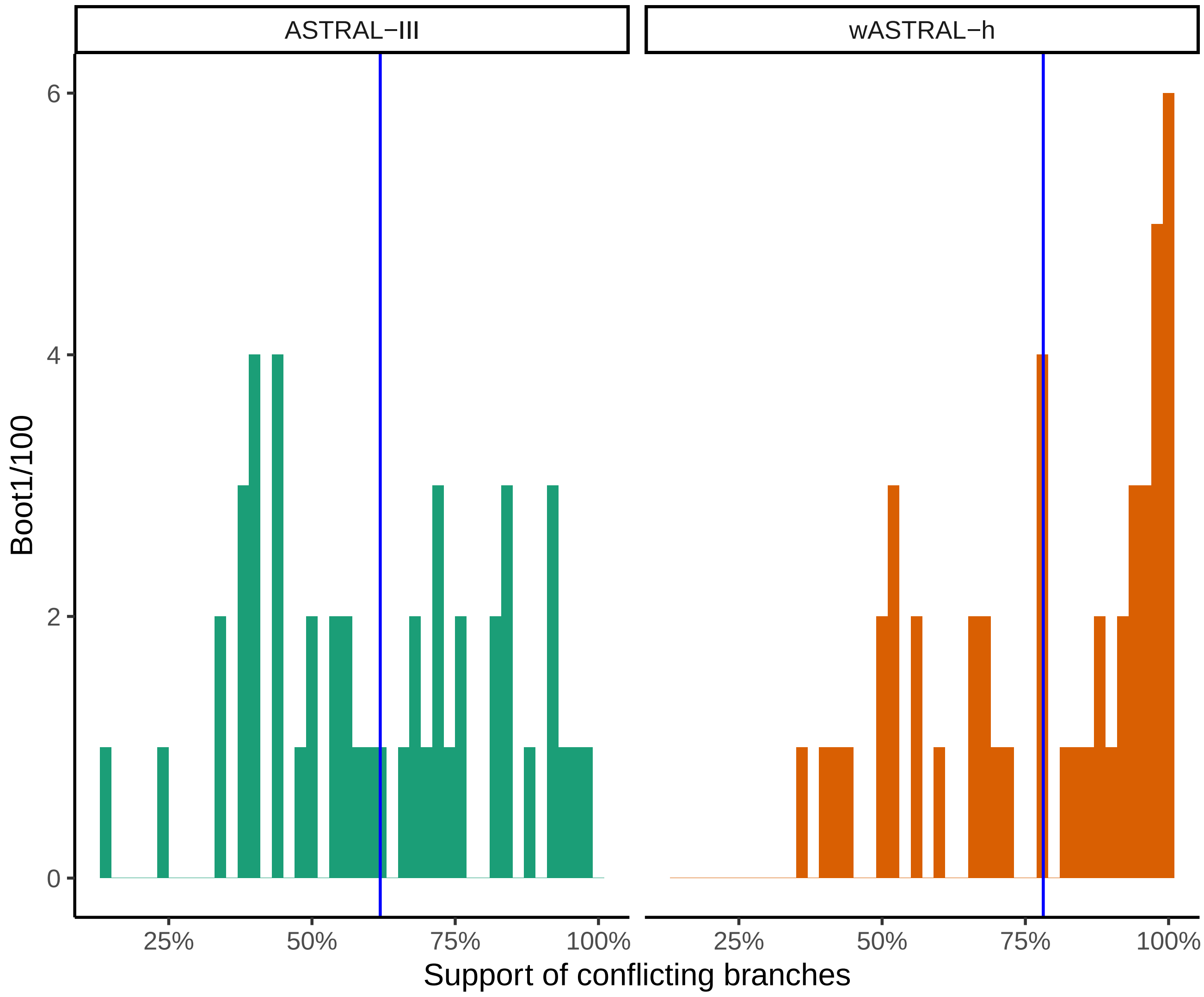

FIG. S13. The distribution of support values of conflicting branches between wASTRAL-h and ASTRAL-III on the 1kp dataset. The ASTRAL-III conflicting branches range between 14% and 99.00% with a mean of 62%. The wASTRAL-h conflicting branches range between 37% and 99.98% with a mean of 78%.

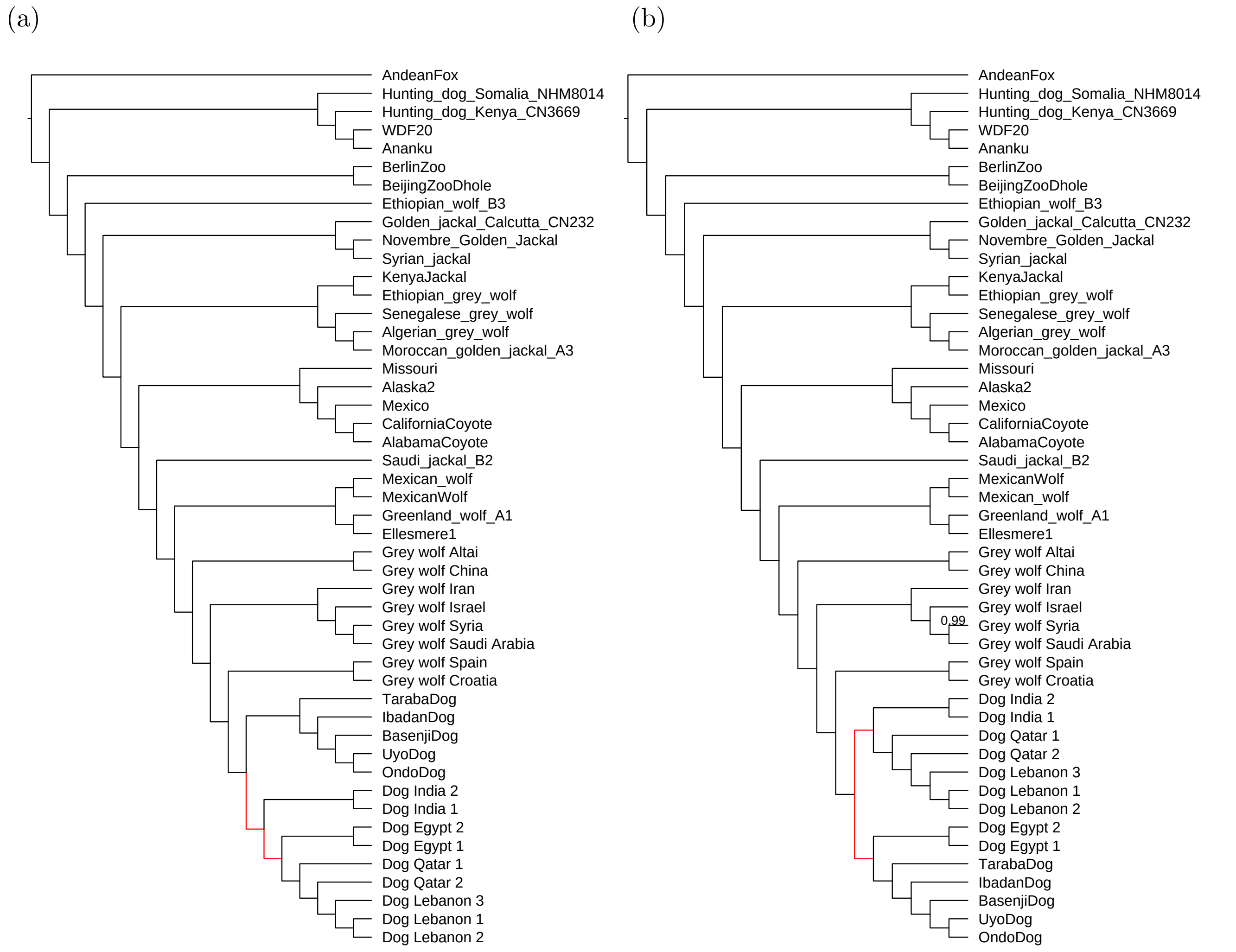

FIG. S14. Inferred species trees (a) from wASTRAL-hybrid with FastTree-2 branch support values as weights using all 459,450 gene trees and (b) from ASTRAL-III using a subset of 100,000 gene trees on canis dataset. Branches support of 100% are omitted.

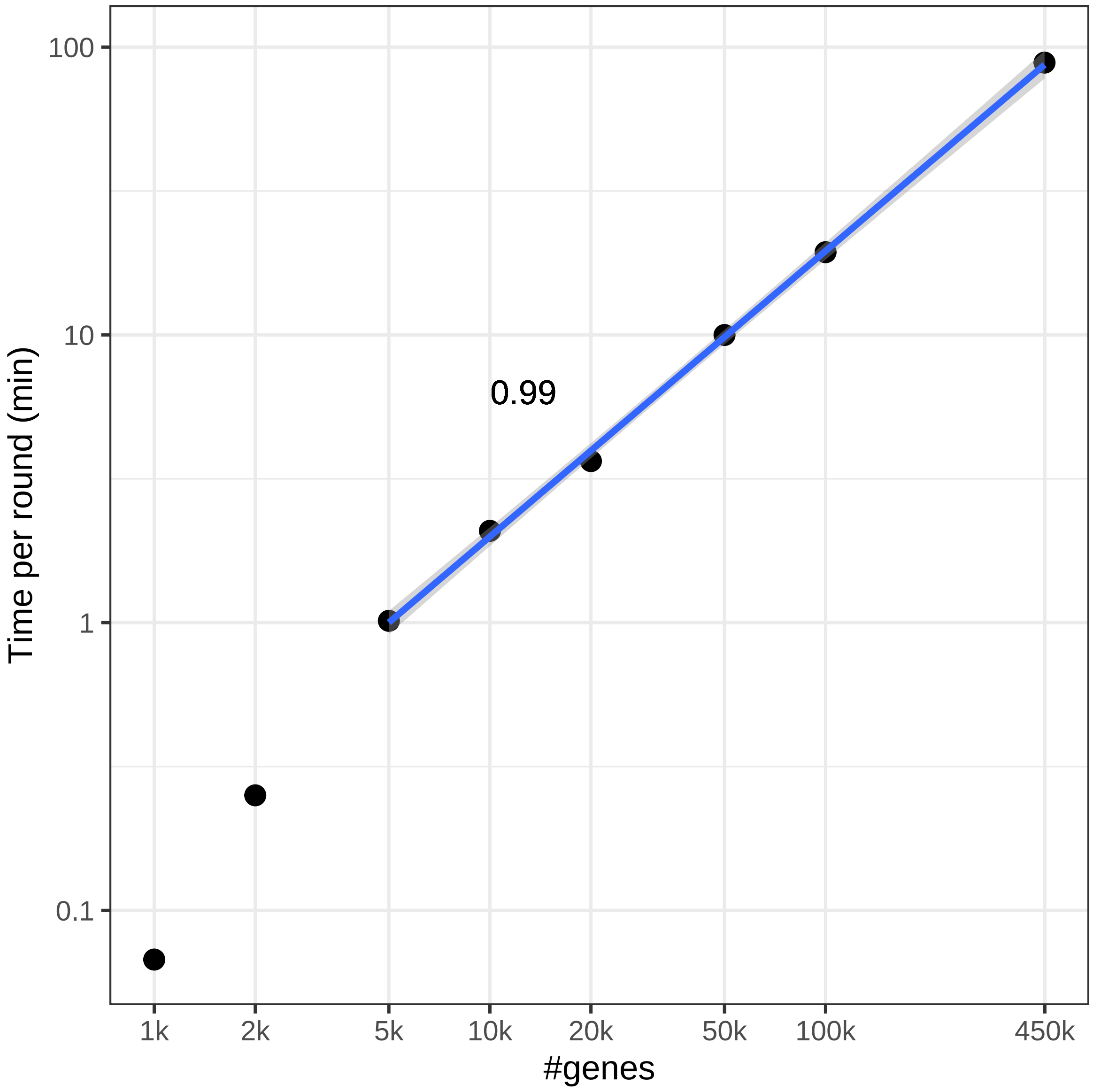

FIG. S15. Normalized time per round of placement by dividing running time by the total number of rounds of placements for wASTRAL-h on the Canis dataset for various k using the new pipeline.

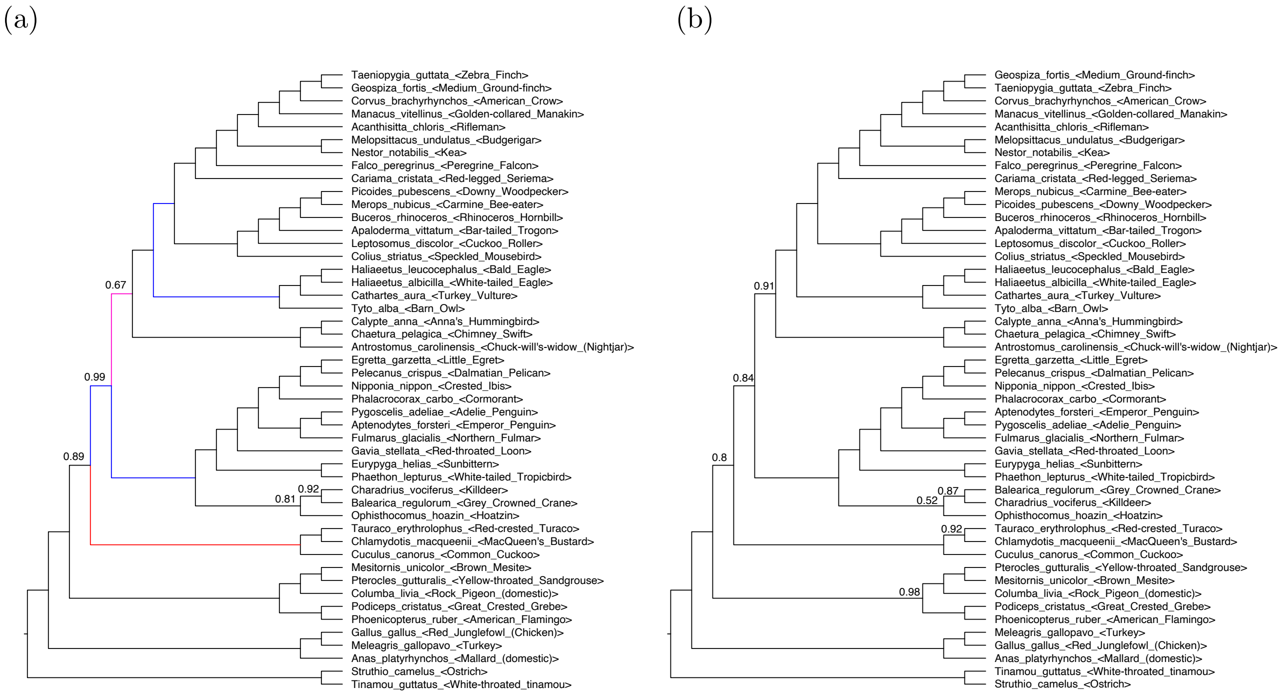

FIG. S16. Inferred species trees from (a) wASTRAL-hybrid with normalized bootstrap support values as weights and (b) ASTRAL-III on gene trees with low (<3% bootstrap) support branches contracted on avian dataset. Branches support of 100% are omitted. Branches that disagree with concatenation (blue), MP-EST binned (red) or both (purple) are identified on the wASTRAL-h tree.

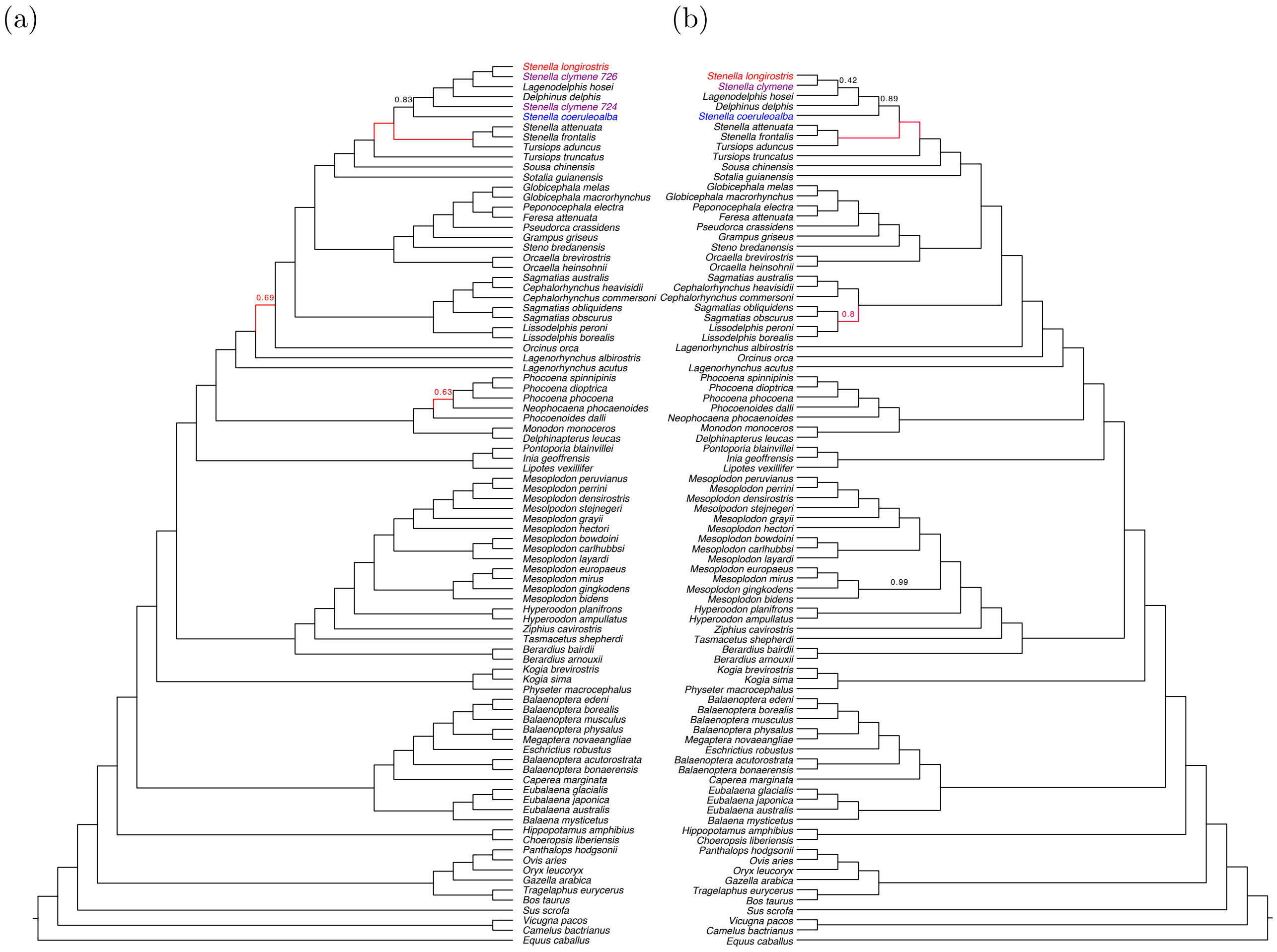

FIG. S17. Inferred species trees from (a) wASTRAL-hybrid with normalized Bayesian support values as weights (with clades of taxa from the same species contracted) and (b) ASTRAL-multi on cetacean dataset. Branches support of 100% are omitted. Branches conflicting with RAxML concatenation are marked red.

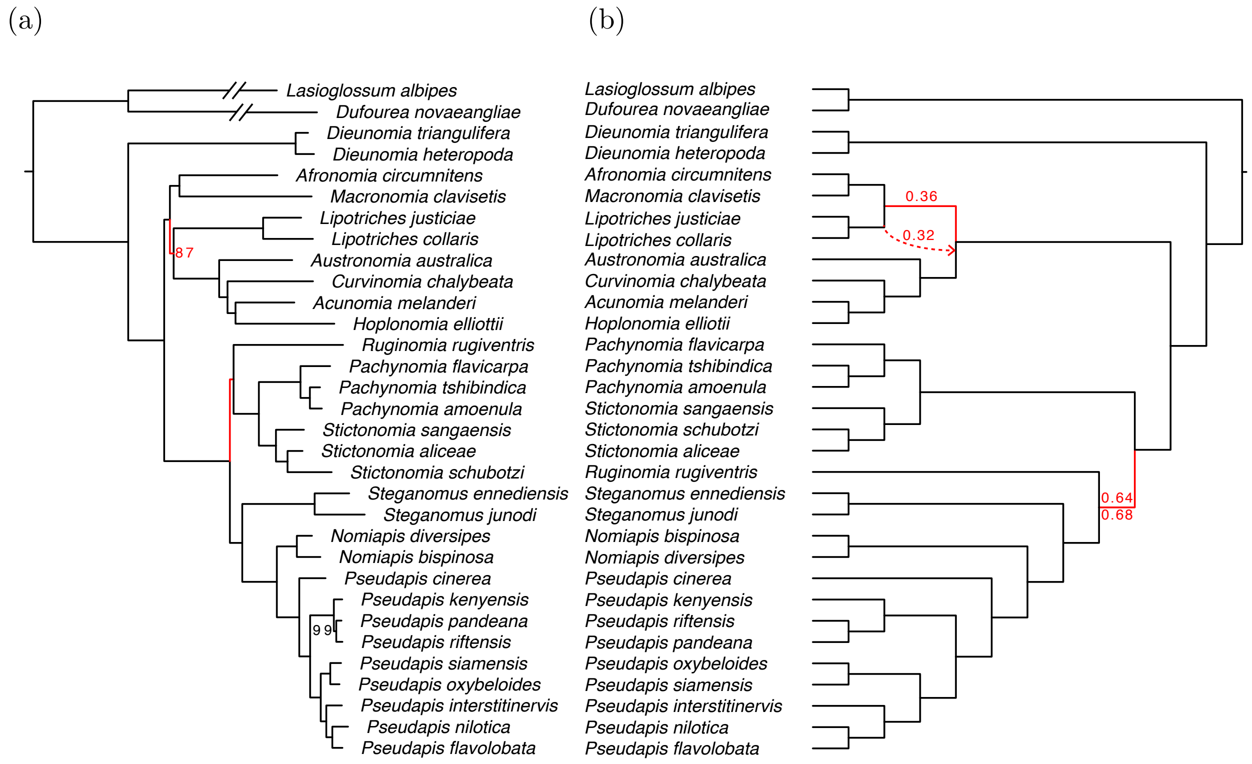

FIG. S18. (a) RAxML on concatenated genes; (b) wASTRAL-hybrid (top and solid red line) and ASTRAL-III (bottom and dashed red line) on Nomiinae dataset.

(a) (b)

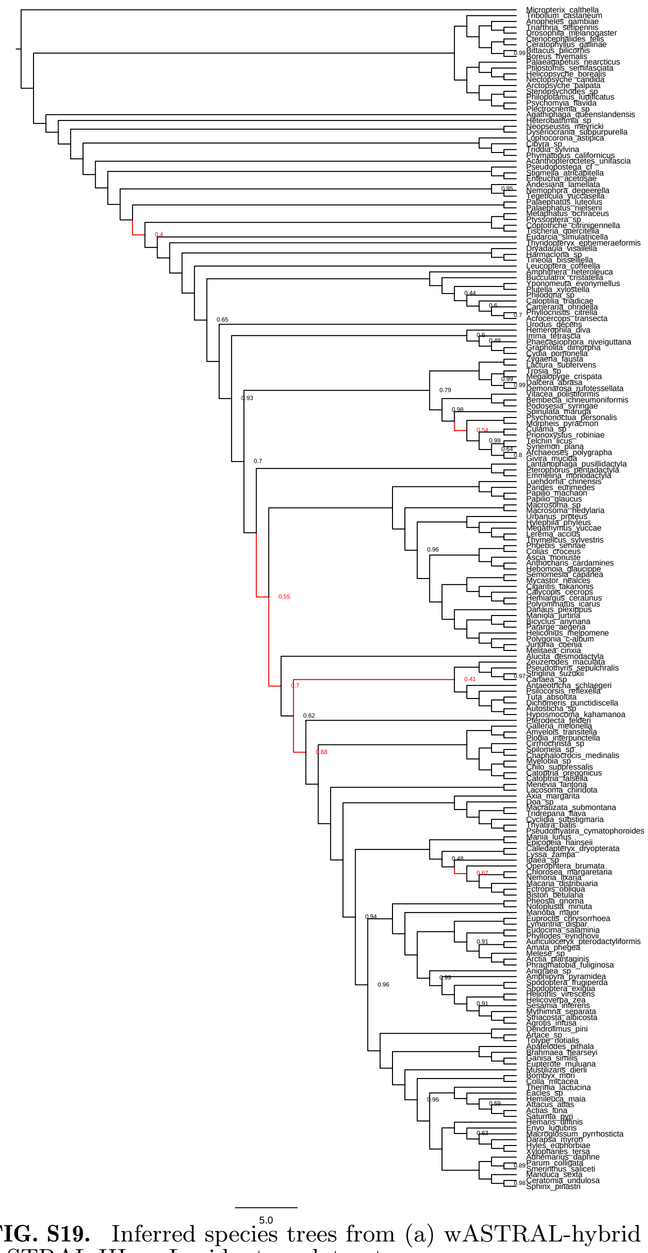

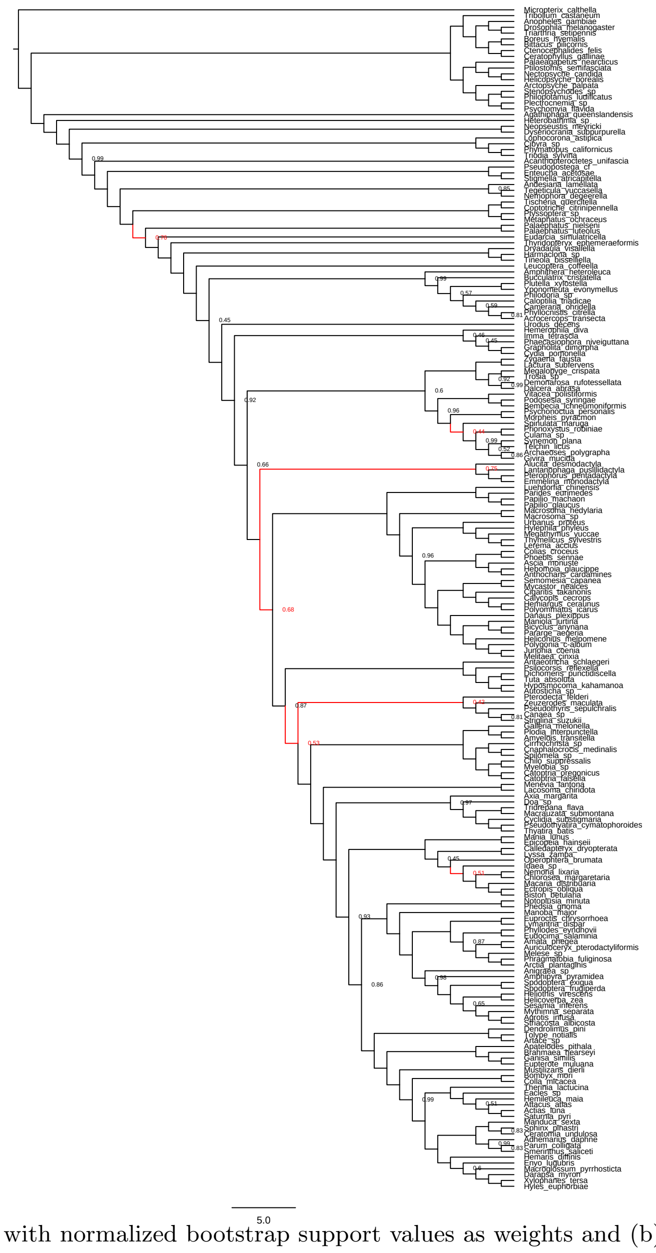

FIG. S19. Inferred species trees from (a) wASTRAL-hybrid with normalized bootstrap support values as weights and (b) ASTRAL-III on Lepidoptera dataset.

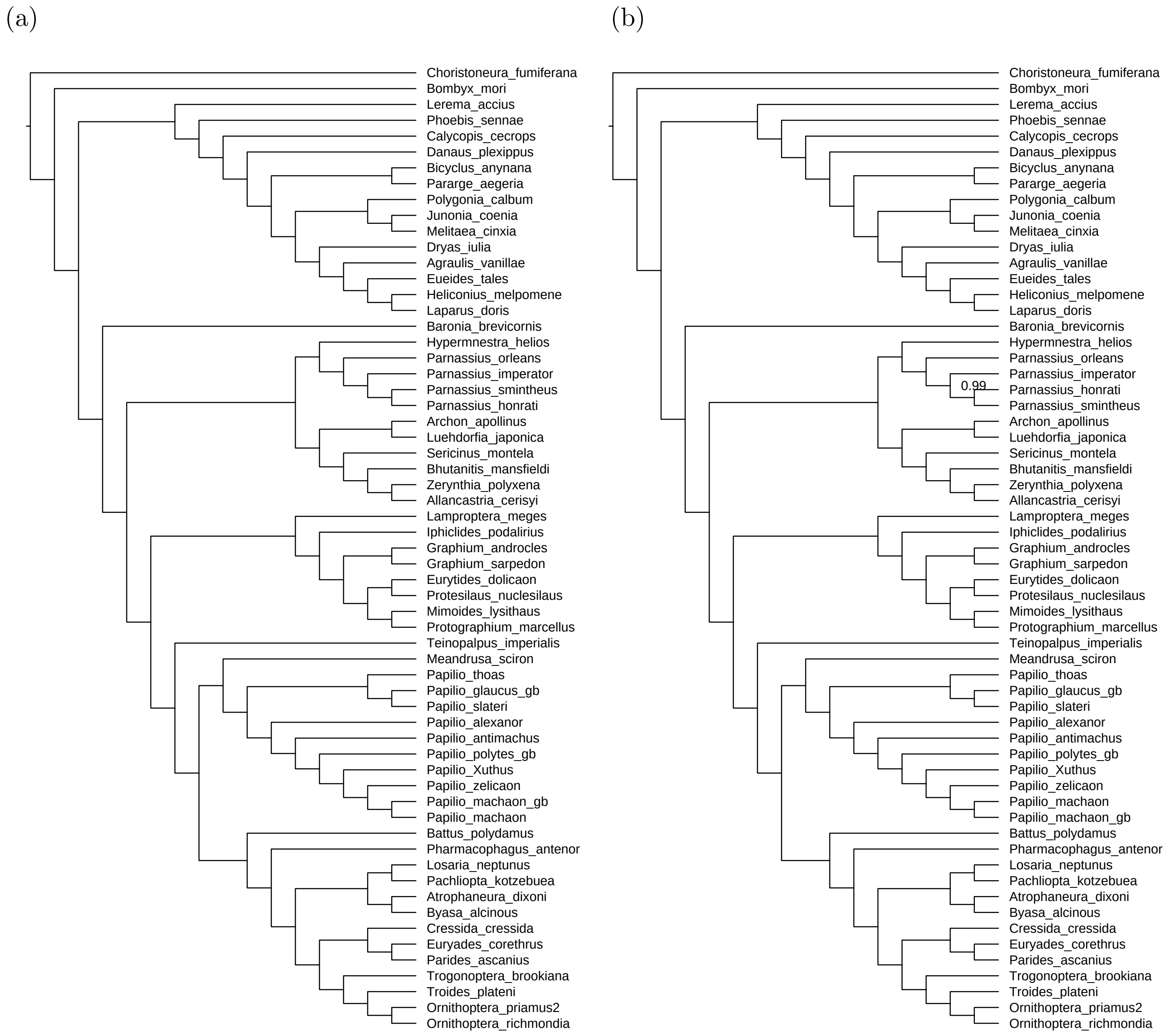

FIG. S20. Inferred species trees from (a) wASTRAL-hybrid with normalized approximate Bayesian support values as weights and (b) ASTRAL-III on Papilionidae dataset.

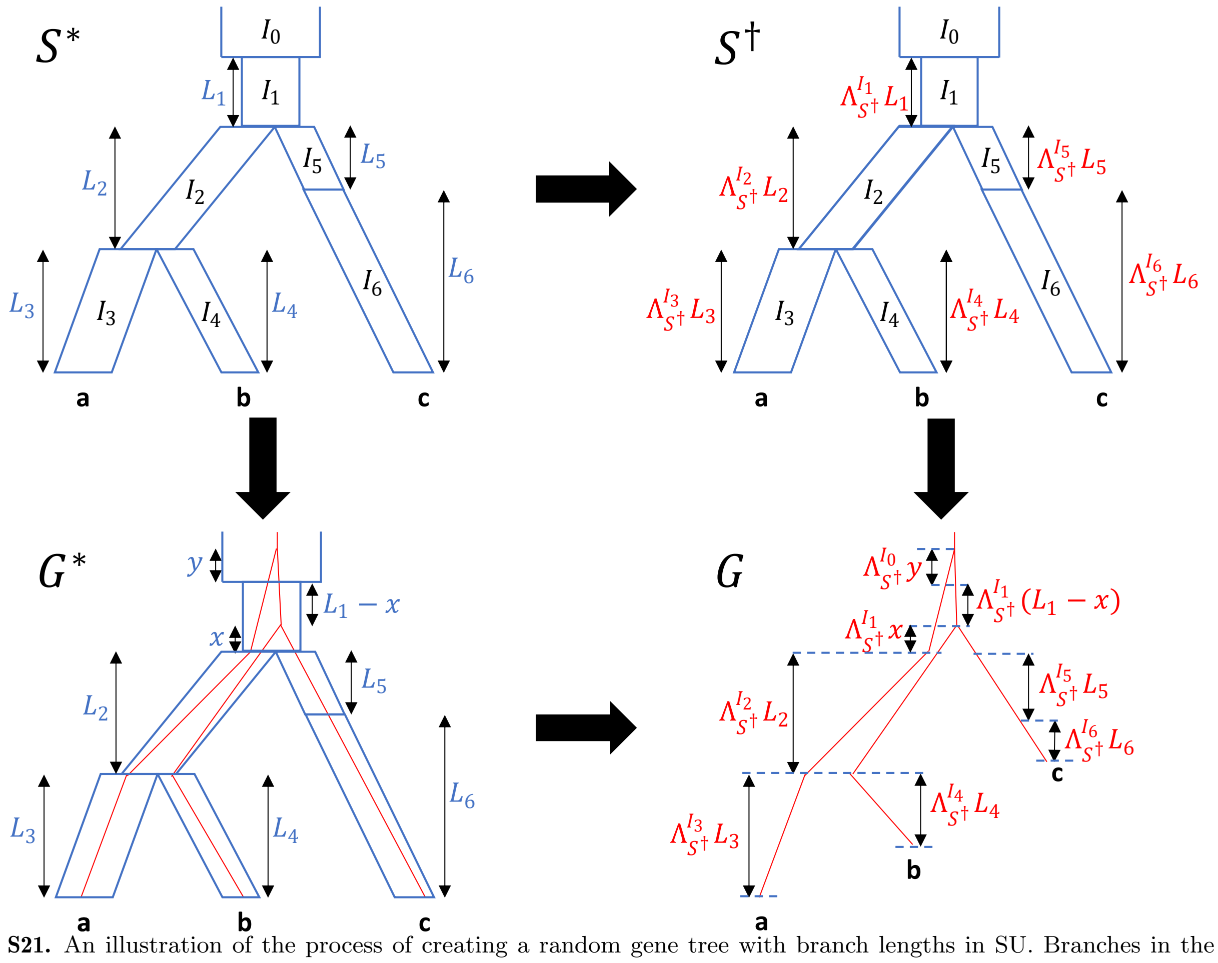

FIG. S21. An illustration of the process of creating a random gene tree with branch lengths in SU. Branches in the true species tree S^* are broken into intervals $I_0 \ldots I_6$. The species tree with SU branch lengths $S^\dagger$ is created by multiplying each branch length in S^* with a corresponding multiplier; the multipliers are jointly drawn from some distribution and are drawn independently across gene trees. Gene tree G^* is sampled under MSC process from S^* independent of $S^\dagger$. However, it inherits the same division of its lineages into segments as S^* at the same locations. The gene tree with SU branch lengths G is created by translating branch lengths of G^* into SU by multiplying the CU length of each of segment I_i by $\Lambda_{S^\dagger}^{I_i}$, the multiplier associated with the segment I_i in $S^\dagger$ and hence G.

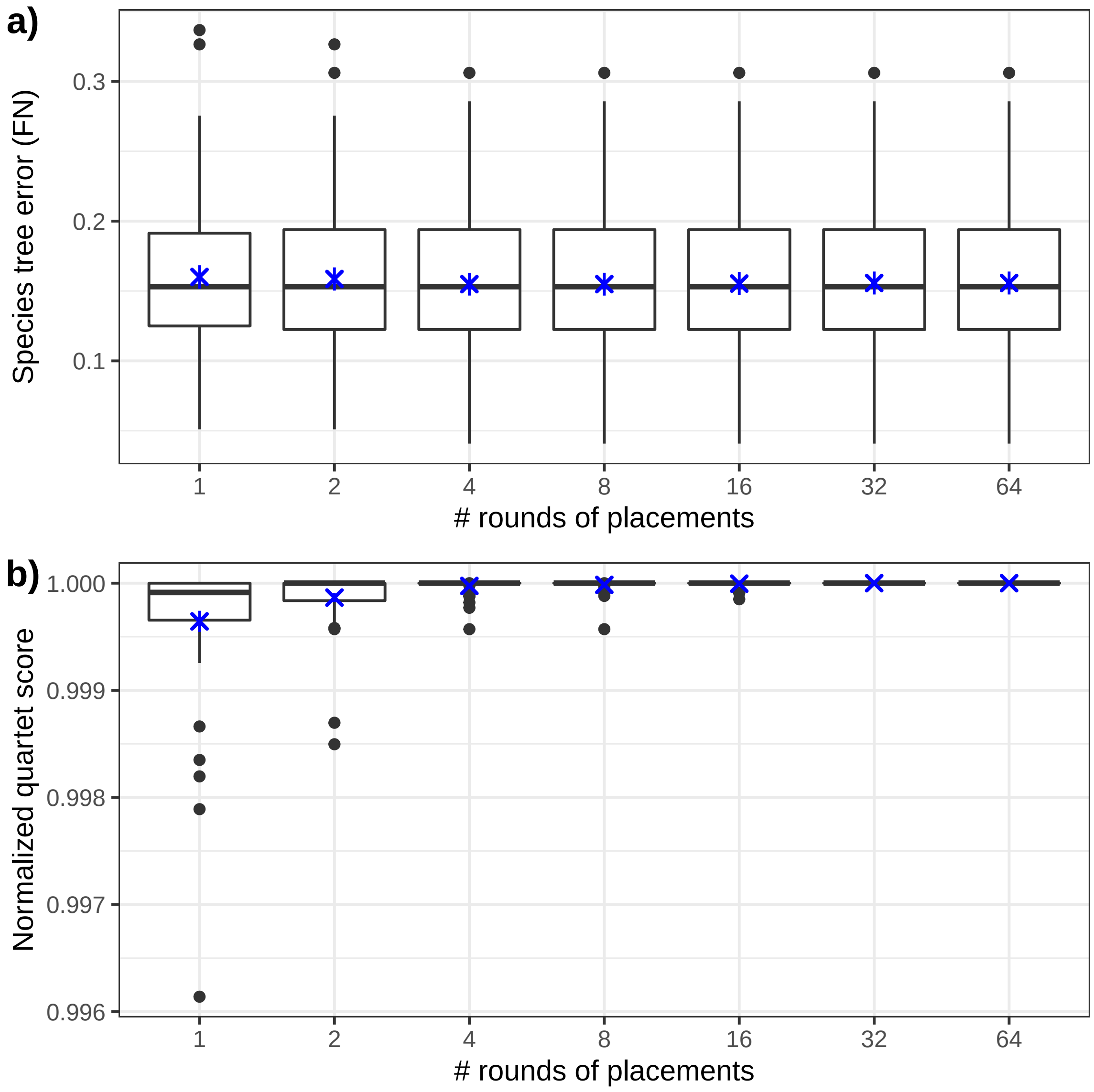

FIG. S22. The species tree estimation error (FN) of wASTRAL-h on S100 dataset as we change the number of rounds of placements in the base algorithm (r). The most difficult case where gene length=200 and k=50 is selected. Mean and standard error (50 replicates) are shown in blue.

Supplementary Algorithm

Algorithm S1 Recursive placement algorithm. `Place` inserts the species i into an existing species tree S and computes tripartition scores $W(A|B|C,\mathcal{G}) := \sum_{G\in\mathcal{G}} W(A\cap\mathcal{L}_G|B\cap\mathcal{L}_G|C\cap\mathcal{L}_G,G)$ for all tripartitions resulting from adding i onto each branch of S. A global counter Q and a set of per-node counters $w_A,w_B,w_C,w^+_{..},w^-_{..},w_{..|.},w_{..|..}$ are all initialized to 0. `OptimalTreeDP` is defined in Algorithm S2. Each gene tree is rooted on an arbitrary branch e and the support of e is kept for the branch on one side of the root and zero support is given to the branch on the other side of root. $\mathcal{L}_v$ is the set of leaves under v.

procedure PLACE($i,S,\mathcal{G}$) ▷ Places species i on tree S according to $\mathcal{G}$
    $W \leftarrow$ empty lookup table ▷ global variables
    COLORLEAFSET($\mathcal{L}_S$,C,$\emptyset$,$\mathcal{G}$,W) ▷ Color all leaves of S as C
    COLORLEAFSET($\{i\}$,B,$\emptyset$,$\mathcal{G}$,W) ▷ Color new species i as B
    COLORNODE(the root of S,i,S,$\mathcal{G}$,W) ▷ Traverse S bottom up
    $O \leftarrow$ OPTIMALTREEDP($\mathcal{L}_S\cup\{i\},\mathcal{L}_S\cup\{i\},W$)
    return (W,O, edge of S onto which i is added to get O)
procedure COLORLEAFSET($\mathcal{L}^*,X,T,\mathcal{G},W$) ▷ Condition: Coloring $\mathcal{L}^*$ as X should match T
    for $G\in\mathcal{G}$ **do**
        for $j\in\mathcal{L}^*\cap\mathcal{L}_G$ **do**
            $W[T] \leftarrow$ UPDATECOUNTERS(leaf node corresponding to j in g, X)
procedure COLORNODE($w,i,S,\mathcal{G},W$) ▷ On start: i is B, others are C; On exit: w is A, others kept
    if w is a leaf **then**
        COLORLEAFSET($\mathcal{L}_w$,A,$\mathcal{L}_w|\{i\}|\mathcal{L}_S-\mathcal{L}_w$,$\mathcal{G}$,$W$)
    else
        $(u,v) := ($ the larger child of w, the smaller child of w $)$
        COLORNODE($v,i,S,\mathcal{G},W$) ▷ Recurse on v, the smaller child
        COLORLEAFSET($\mathcal{L}_v$,C,$\emptyset$,$\mathcal{G}$,W) ▷ Undo coloring of v to enable recursing on u.
        COLORNODE($u,i,S,\mathcal{G},W$) ▷ Recurse on u, the large child
        COLORLEAFSET($\mathcal{L}_v$,B,$\mathcal{L}_u|\{i\}\cup\mathcal{L}_v|\mathcal{L}_S-\mathcal{L}_w$,$\mathcal{G}$,$W$) ▷ Tripartition of w when adding i above v
        COLORLEAFSET($\{i\}$,A,$\{i\}\cup\mathcal{L}_u|\mathcal{L}_v|\mathcal{L}_S-\mathcal{L}_w$,$\mathcal{G}$,$W$) ▷ Tripartition of w when adding i above u
        COLORLEAFSET($\{i\}$,C,$\mathcal{L}_u|\mathcal{L}_v|\{i\}\cup\mathcal{L}_S-\mathcal{L}_w$,$\mathcal{G}$,$W$) ▷ Tripartition of w when adding i above w
        COLORLEAFSET($\{i\}$,B,$\emptyset$,$\mathcal{G}$,W)
        COLORLEAFSET($\mathcal{L}_v$,A,$\mathcal{L}_w|\{i\}|\mathcal{L}_S-\mathcal{L}_w$,$\mathcal{G}$,$W$) ▷ Tripartition of the new parent of i and w
procedure RECURSIVEUPDATE(w)
    $(u,v,e) := ($ the left child of w, the right child of w, the parent branch of w $)$
    for $(X,Y,Z)\in\{(A,B,C),(B,C,A),(C,A,B)\}$ **do**
        $Q \leftarrow Q - w_{XX|YZ}$
        $w_{XX|YZ} \leftarrow v_X u_{YZ|X} + u_X v_{YZ|X} + u_{XX|Z} v_Y + v_{XX|Z} u_Y + u_{XX|Y} v_Z + v_{XX|Y} u_Z$
            $+(u^+_{YZ} v^+_{XX} - u^-_{YZ} v^-_{XX}) + (u^+_{XX} v^+_{YZ} - u^-_{XX} v^-_{YZ})$
        $Q \leftarrow Q + w_{XX|YZ}$
        if w is not the root **then**
            $(w_X,w_Y,w_Z) \leftarrow \big((u_X+v_X)e^{-l(e)},(u_Y+v_Y)e^{-l(e)},(u_Z+v_Z)e^{-l(e)}\big)$
            $w^+_{XX} \leftarrow u^+_{XX} + v^+_{XX} + u_X v_X$
            $w^-_{XX} \leftarrow (u^-_{XX} + v^-_{XX} + u_X v_X)\big(1-s(e)\big)$
            $w^+_{YZ} \leftarrow u^+_{YZ} + v^+_{YZ} + u_Y v_Z + u_Z v_Y$
            $w^-_{YZ} \leftarrow (u^-_{YZ} + v^-_{YZ} + u_Y v_Z + u_Z v_Y)\big(1-s(e)\big)$
            $w_{YZ|X} \leftarrow \big(u_{YZ|X} + v_{YZ|X} + (u^+_{YZ} - u^-_{YZ})v_X + u_X(v^+_{YZ} - v^-_{YZ})\big)e^{-l(e)}$
            $w_{XX|Y} \leftarrow \big(u_{XX|Y} + v_{XX|Y} + (u^+_{XX} - u^-_{XX})v_Y + u_Y(v^+_{XX} - v^-_{XX})\big)e^{-l(e)}$
            $w_{XX|Z} \leftarrow \big(u_{XX|Z} + v_{XX|Z} + (u^+_{XX} - u^-_{XX})v_Z + u_Z(v^+_{XX} - v^-_{XX})\big)e^{-l(e)}$
            RECURSIVEUPDATE(the parent of w)
procedure UPDATECOUNTERS(w,X) ▷ w is a leaf, X is a color
    $e :=$ the parent branch of w
    $(w_A,w_B,w_C) \leftarrow (0,0,0)$
    $w_X \leftarrow e^{-l(e)}$
    RECURSIVEUPDATE(the parent of w)
    return Q

Algorithm S2 The Algorithm S2 of $O(n^2 kH \log n)$ running time. At start, the function is called as with $\mathcal{L}_S, \mathcal{G}, r$ as input.

procedure NAIVEPLACEMENT($T, \mathcal{G}, r$)
  $W^* \leftarrow$ empty lookup table from tripartitions to their weights
  for $i \in \{1,...,r\}$ **do**
    shuffle T
    $S_i \leftarrow$ tree with leaves T_1, T_2, and T_3
    for $j \in \{4,...,|T|\}$ **do**
      $W_i, S_i, e \leftarrow$ PLACE($T_j, S_i, \mathcal{G}$)
    Add all elements of W_i to W^*
  return OPTIMALTREEDP(T, T, W^*)
procedure OPTIMALTREEDP($P, \mathcal{L}, W$)
  if DPTree(P) available **then**
    return DPTree(P)
  if $|P| = 1$ **then**
    DPScore(P) $\leftarrow 0$
    DPTree(P) $\leftarrow$ Singleton rooted tree with leafset P
  else
    $X \leftarrow -\infty$
    for $A \in \{A : W[A|P-A|\mathcal{L}-P]$ has been computed$\}$ **do**
      $S_1 \leftarrow$ OPTIMALTREEDP($A, \mathcal{L}, W$)
      $S_2 \leftarrow$ OPTIMALTREEDP($P-A, \mathcal{L}, W$)
      if DPScore(A) + DPScore($P-A$) + $W[A|P-A|\mathcal{L}-P] > X$ **then**
        $X \leftarrow$ DPScore(A) + DPScore($P-A$) + $W[A|P-A|\mathcal{L}-P]$
        DPTree(P) $\leftarrow$ merge subtrees S_1 and S_2 at root
    DPScore(P) $\leftarrow X$
  return DPTree(P)

Algorithm S3 The DAC algorithm of $O(n^{1.5+\epsilon}k)$ running time given some assumptions. OptimalTreeDP and NaivePlacement are defined in Algorithm S2, and Place is defined in Algorithm S1. At start, the function is called as with $\mathcal{L}_S, \mathcal{G}, r$ as input.

procedure TwoStepPlacement($T, \mathcal{G}, r$)
  $W^* \leftarrow$ empty lookup table from tripartitions to their weights
  for $i \in \{1, ..., r\}$ **do**
    $T_i \leftarrow$ a subsample of T by removing each element independently with probability $1 - 1/\sqrt{|T|}$
    $S_i :=$ NaivePlacement($T_i, \mathcal{G}, \sqrt{|T|}$)
    for $e \in E_{S_i}$ **do**
      $C_e \leftarrow$ empty list
    for $j \in T - T_i$ **do**
      $W, S_o, e \leftarrow$ Place($j, S_i, \mathcal{G}$)
      add T_j to C_e
    $C_\emptyset \leftarrow$ empty list
    $S'_i \leftarrow S_i$
    for $e \in$ branches of S_i **do**
      $S_e \leftarrow S_i$
      for $j \in C_e$ **do**
        $W, S_o, e' \leftarrow$ Place($j, S_e, \mathcal{G}$)
        if $e' \in S_i - \{e\}$ **then**
          add j to $C_\emptyset$
        else
          $S_e \leftarrow S_o$
      $S'_i \leftarrow$ The merger of compatible trees S_e and S'_i
    for $j \in C_\emptyset$ **do**
      $W_i, S'_i, e \leftarrow$ Place($j, S'_i, \mathcal{G}$)
    if $C_\emptyset = \emptyset$ **then**
      $W_i, S'_i, e \leftarrow$ Place($\emptyset, S'_i, \mathcal{G}$)
    Add all elements of W_i to W^*
  return OptimalTreeDP(T, T, W^*)

Proofs

Weighting by support: Proof of Proposition 1 and Theorem 1

For ease of reference, we reproduce Table 2 from the main paper here:

$\mathbb{E}\big[(\cdot)(\cdot)\big\|\alpha_{G,Q}\big]$	$\delta_G(ab\|cd)$	$\delta_G(ac\|bd)$
$\delta_{G^*}(ab\|cd)$	$\geq\frac{1}{3}(1+2\theta_Q)\big(\alpha_{G,Q}+\frac{1}{3}(1-\alpha_{G,Q})(1-\beta_Q)\big)$	$\leq\frac{1}{3}(1+2\theta_Q)\big(\frac{1}{3}(1-\alpha_{G,Q})(1+\beta_Q)\big)$
$\delta_{G^*}(ac\|bd)$	$\geq\frac{1}{3}(1-\theta_Q)\big(\frac{1}{3}(1-\alpha_{G,Q})(1-\beta_Q)\big)$	$\leq\frac{1}{3}(1-\theta_Q)\big(\alpha_{G,Q}+\frac{1}{3}(1-\alpha_{G,Q})(1+\beta_Q)\big)$
$\delta_{G^*}(ad\|bc)$	$\geq\frac{1}{3}(1-\theta_Q)\big(\frac{1}{3}(1-\alpha_{G,Q})(1-\beta_Q)\big)$	$\leq\frac{1}{3}(1-\theta_Q)\big(\frac{1}{3}(1-\alpha_{G,Q})(1+\beta_Q)\big)$
$\mathbb{E}\big[(\cdot)(\cdot)\big\|\alpha_{G,Q}\big]$	$w_G(ab\|cd)$	$w_G(ac\|bd)$
$\delta_{G^*}(ab\|cd)$	$\geq\frac{1}{3}(1+2\theta_Q)\big(\alpha_{G,Q}+\frac{1}{3}(1-\alpha_{G,Q})(1-\beta_Q)\big)^2$	$\leq\frac{1}{3}(1+2\theta_Q)\big(\frac{1}{3}(1-\alpha_{G,Q})(1+\beta_Q)\big)^2$
$\delta_{G^*}(ac\|bd)$	$\geq\frac{1}{3}(1-\theta_Q)\big(\frac{1}{3}(1-\alpha_{G,Q})(1-\beta_Q)\big)^2$	$\leq\frac{1}{3}(1-\theta_Q)\big(\alpha_{G,Q}+\frac{1}{3}(1-\alpha_{G,Q})(1+\beta_Q)\big)^2$
$\delta_{G^*}(ad\|bc)$	$\geq\frac{1}{3}(1-\theta_Q)\big(\frac{1}{3}(1-\alpha_{G,Q})(1-\beta_Q)\big)^2$	$\leq\frac{1}{3}(1-\theta_Q)\big(\frac{1}{3}(1-\alpha_{G,Q})(1+\beta_Q)\big)^2$

Recall that the expected value and variance of $\alpha_{G,Q}$ across genes is denoted by $\bar{\alpha_Q}$ and σ_α^2.

PROPOSITION 1. *For each estimated gene tree G,* $\mathbb{E}\big[\delta_G(ab|cd)-\delta_G(ac|bd)\big]\geq\theta_Q\bar{\alpha_Q}-\frac{2}{3}(1-\bar{\alpha_Q})\beta_Q$ *and* $\mathbb{E}\big[w_G(ab|cd)-w_G(ac|bd)\big]\geq\frac{1}{9}\theta_Q(3+2\beta_Q)(\bar{\alpha_Q}^2+\sigma_\alpha^2)+\frac{2}{9}(3-\beta_Q)\theta_Q\bar{\alpha_Q}-\frac{4}{9}(1-\bar{\alpha_Q})\beta_Q$.

Proof. To prove the Proposition, we start with the following lemma.

LEMMA 1. *For each estimated gene tree G with a given $\alpha_{G,Q}$,*

$$\mathbb{E}\big[\delta_G(ab|cd)-\delta_G(ac|bd)\big|\alpha_{G,Q}\big]\geq\theta_Q\alpha_{G,Q}-\frac{2}{3}(1-\alpha_{G,Q})\beta_Q$$

and

$$\mathbb{E}\big[w_G(ab|cd)-w_G(ac|bd)\big|\alpha_{G,Q}\big]\geq\frac{1}{9}(3\alpha_{G,Q}-2\beta_Q+2\alpha_{G,Q}\beta_Q+6)\theta_Q\alpha_{G,Q}-\frac{4}{9}(1-\alpha_{G,Q})\beta_Q\,.$$

Proof. From Table 2, we can compute

$$\begin{aligned}&\mathbb{E}\big[\delta_G(ab|cd)-\delta_G(ac|bd)\big|\alpha_{G,Q}\big]\\&=\mathbb{E}\Big[\big(\delta_G(ab|cd)-\delta_G(ac|bd)\big)\big(\delta_{G^*}(ab|cd)+\delta_{G^*}(ac|bd)+\delta_{G^*}(ad|bc)\big)\Big|\alpha_{G,Q}\Big]\\&\geq\frac{1}{3}\big((1+2\theta_Q)\alpha_{G,Q}+\frac{1}{3}(1-\alpha_{G,Q})(1-\beta_Q)\big)-\frac{1}{3}\big((1-\theta_Q)\alpha_{G,Q}+\frac{1}{3}(1-\alpha_{G,Q})(1+\beta_Q)\big)\\&=\theta_Q\alpha_{G,Q}-\frac{2}{3}(1-\alpha_{G,Q})\beta_Q\,;\end{aligned}$$

similarly,

$$\begin{aligned}&\mathbb{E}\big[w_G(ab|cd)-w_G(ac|bd)\big|\alpha_{G,Q}\big]\\&=\mathbb{E}\Big[\big(w_G(ab|cd)-w_G(ac|bd)\big)\big(\delta_{G^*}(ab|cd)+\delta_{G^*}(ac|bd)+\delta_{G^*}(ad|bc)\big)\Big|\alpha_{G,Q}\Big]\\&\geq\frac{1}{3}(1+2\theta_Q)\alpha_{G,Q}\big(\alpha_{G,Q}+\frac{2}{3}(1-\alpha_{G,Q})(1-\beta_Q)\big)+\big(\frac{1}{3}(1-\alpha_{G,Q})(1-\beta_Q)\big)^2\\&\quad-\frac{1}{3}(1-\theta_Q)\alpha_{G,Q}\big(\alpha_{G,Q}+\frac{2}{3}(1-\alpha_{G,Q})(1+\beta_Q)\big)-\big(\frac{1}{3}(1-\alpha_{G,Q})(1+\beta_Q)\big)^2\\&\geq\frac{1}{9}(3\alpha_{G,Q}-2\beta_Q+2\alpha_{G,Q}\beta_Q+6)\theta_Q\alpha_{G,Q}-\frac{4}{9}(1-\alpha_{G,Q})\beta_Q\,.\end{aligned}$$

□

From this lemma, we can prove the proposition. First, assume $\alpha_{G,Q}$ is drawn from a discrete distribution. Then,

$$\begin{aligned}\mathbb{E}\big[\delta_G(ab|cd)-\delta_G(ac|bd)\big] &= \sum_{\alpha_{G,Q}} \mathbb{E}\big[\delta_G(ab|cd)-\delta_G(ac|bd)\big|\alpha_{G,Q}\big]\mathrm{P}(\alpha_{G,Q}) \\ &\geq \sum_{\alpha_{G,Q}} \big(\theta_Q\alpha_{G,Q}-\frac{2}{3}(1-\alpha_{G,Q})\beta_Q\big)\mathrm{P}(\alpha_{G,Q}) = \theta_Q\bar{\alpha_Q}-\frac{2}{3}(1-\bar{\alpha_Q})\beta_Q\end{aligned}$$

and

$$\begin{aligned}&\mathbb{E}\big[w_G(ab|cd)-w_G(ac|bd)\big] = \sum_{\alpha_{G,Q}} \mathbb{E}\big[w_G(ab|cd)-w_G(ac|bd)\big|\alpha_{G,Q}\big]\mathrm{P}(\alpha_{G,Q}) \\ &\geq \sum_{\alpha_{G,Q}} \big(\frac{1}{9}(3\alpha_{G,Q}-2\beta_Q+2\alpha_{G,Q}\beta_Q+6)\theta_Q\alpha_{G,Q}-\frac{4}{9}(1-\alpha_{G,Q})\beta_Q\big)\mathrm{P}(\alpha_{G,Q}) \\ &= \frac{1}{9}\theta_Q(3+2\beta_Q)\mathbb{E}[\alpha_{G,Q}^2]+\frac{2}{9}(3-\beta_Q)\theta_Q\bar{\alpha_Q}-\frac{4}{9}(1-\bar{\alpha_Q})\beta_Q \\ &= \frac{1}{9}\theta_Q(3+2\beta_Q)(\bar{\alpha_Q}^2+\sigma_\alpha^2)+\frac{2}{9}(3-\beta_Q)\theta_Q\bar{\alpha_Q}-\frac{4}{9}(1-\bar{\alpha_Q})\beta_Q\,.\end{aligned}$$

It is straightforward to change these calculations to use integral instead of sum and $\mathrm{P}(\alpha_{G,Q})$ to the PDF in the case that the distribution of $\alpha_{G,Q}$ is continuous. □

Theorem 1. *Given estimated gene trees furnished with support generated under MSC+Error+Support model, there exist conditions where (3) guarantee a statistically consistent estimator of S^* but (2) does not, and the reverse is not true.*

Proof. Recall that (1) states

$$W(S,\mathcal{G}) := \sum_{G\in\mathcal{G}}\sum_{Q\in\mathcal{Q}(S)} w_G(S\upharpoonright Q)\,.$$

It means that in order to produce a statistically consistent estimator using (1), the following equation must be satisfied for the true species tree topology S^* and any species tree topology S:

$$\mathbb{E}\big[W(S^*,\mathcal{G})-W(S,\mathcal{G})\big] = |\mathcal{G}|\sum_{Q\in\mathcal{Q}(S)}\mathbb{E}\big[w_G(S^*\upharpoonright Q)-w_G(S\upharpoonright Q)\big]\geq 0 \tag{9}$$

Notice that proving for any quartet $Q=\{a,b,c,d\}$ we have $\mathbb{E}\big[w_G(ab|cd)-w_G(ac|bd)\big]\geq 0$ and $\mathbb{E}\big[w_G(ab|cd)-w_G(ad|bc)\big]\geq 0$ where $S^*\upharpoonright Q=ab|cd$ is sufficient to prove (9); on the other hand, proving for any quartet $Q=\{a,b,c,d\}$ where the internal branch of $S^*\upharpoonright Q$ corresponds to only one branch in S^*, we have $\mathbb{E}\big[w_G(ab|cd)-w_G(ac|bd)\big]\geq 0$ and $\mathbb{E}\big[w_G(ab|cd)-w_G(ad|bc)\big]\geq 0$ where $S^*\upharpoonright Q=ab|cd$ is necessary to prove (9).

Thus, from Proposition 1, we have guaranteed statistical consistency for wASTRAL for support under

$$D=\bigcap_{Q\in\mathcal{Q}(S)}\big\{(\theta_Q,\bar{\alpha_Q},\sigma_\alpha,\beta_Q)\in[0,1]^4 : \frac{1}{9}\theta_Q(3+2\beta_Q)(\bar{\alpha_Q}^2+\sigma_\alpha^2)+\frac{2}{9}(3-\beta_Q)\theta_Q\bar{\alpha_Q}-\frac{4}{9}(1-\bar{\alpha_Q})\beta_Q\geq 0\big\}\,.$$

Similarly, we have guaranteed statistical consistency for unweighted ASTRAL under

$$D'=\bigcap_{Q\in\mathcal{Q}(S)}\big\{(\theta_Q,\bar{\alpha_Q},\sigma_\alpha,\beta_Q)\in[0,1]^4 : \theta_Q\bar{\alpha_Q}-\frac{2}{3}(1-\bar{\alpha_Q})\beta_Q\geq 0\big\}\,.$$

To prove Theorem 1, we only need to prove that D' is a proper subset of D.

We can prove $D' \subseteq D$, as for any Q, if $(\theta_Q, \bar{\alpha_Q}, \sigma_\alpha, \beta_Q) \in [0,1]^4$ and $\theta_Q \bar{\alpha_Q} - \frac{2}{3}(1-\bar{\alpha_Q})\beta_Q \geq 0$, then

$$\begin{aligned}&\frac{1}{9}\theta_Q(3+2\beta_Q)(\bar{\alpha_Q}^2+\sigma_\alpha^2)+\frac{2}{9}(3-\beta_Q)\theta_Q\bar{\alpha_Q}-\frac{4}{9}(1-\bar{\alpha_Q})\beta_Q\\ &=\frac{1}{9}\theta_Q(3+2\beta_Q)\sigma_\alpha^2+\frac{1}{3}\theta_Q(1-\theta_Q)\bar{\alpha_Q}^2+(\frac{1}{3}\theta_Q\bar{\alpha_Q}+\frac{2}{3})\big(\theta_Q\bar{\alpha_Q}-\frac{2}{3}(1-\bar{\alpha_Q})\beta_Q\big)\geq 0\,.\end{aligned}$$

We can also prove $D' \neq D$, as if for some Q, $\theta_Q = 0.25, \bar{\alpha_Q} = 0.5, \beta_Q = 0.4$,

$$\theta_Q\bar{\alpha_Q}-\frac{2}{3}(1-\bar{\alpha_Q})\beta_Q=-\frac{1}{120}<0$$

and

$$\frac{1}{9}\theta_Q(3+2\beta_Q)(\bar{\alpha_Q}^2+\sigma_\alpha^2)+\frac{2}{9}(3-\beta_Q)\theta_Q\bar{\alpha_Q}-\frac{4}{9}(1-\bar{\alpha_Q})\beta_Q=\frac{7}{720}+\frac{19}{180}\sigma_\alpha^2>0\,.$$

Thus D' is a proper subset of D and we conclude the proof.

□

Weighting by length: Proof of Propositions 2 and 3 and Theorem 2

Before providing the proofs, we remind the reader of one property of the coalescent model. According to the coalescent model, at any point along a branch of the species tree with i gene tree lineages, the time (i.e., distance) x to the next coalescent event, reducing the number of lineages to $i-1$, is exponentially distributed with the rate $\binom{i}{2}$, resulting in probability density function (PDF):

$$\frac{i(i-1)}{2}e^{-\frac{i(i-1)}{2}x}, \tag{10}$$

and the two lineages that coalesce are independent of x.

Proposition 2. *For a true quartet species tree S^* with topology $ab|cd$ and input gene trees $\mathcal{G}$ generated under the naive model with any multiplier λ, let f be the distance between anchors of S^*. As $f \to 0$, given $k=\Theta(f^{-2})$ gene trees, we have $Var[X_G]=\Theta_f(1)$ and*

$$\frac{\mathbb{E}[X_G]}{\sqrt{Var[X_G]}}=\frac{1+4\lambda}{1+2\lambda}\sqrt{\frac{3}{2}}f+O(f^2).$$

Proof. We analyze balanced and unbalanced trees separately.

Case 1: Unbalanced trees (i.e., the root of S^* has a terminal branch as a child). W.o.l.g., we assume the root branch is located on branch leading to d.

Let p,q, and r be the MRCA nodes of (a,b), (a,c), and (a,d) on rooted species tree S^*, respectively. Let p' and r' be the points of coalescence of leaves a,b and leaves c,d on the rooted gene tree G, respectively. Let x, y_0, and z be the CU difference in heights of points (p,p'), (q,r), and (r,r'), respectively. Note that f is the length of (p,q). Let $L:=l_{S^*}(a,p)+l_{S^*}(b,p)+l_{S^*}(c,r)+l_{S^*}(d,r)$. Notice that $l_G(a,p)+l_G(b,p)+l_G(c,r)+l_G(d,r)=\lambda L$ and $l_G(a,b)+l_G(c,d)=\lambda(2x+2z+L)$.

Let $f_X(x)$ be the probability density that x is the CU difference in heights of (p,p') and p' is the lowest point of coalescence. Notice that by (10):

$$f_X(x)=\begin{cases} e^{-x} & 0\le x\le f \\ \frac{1}{\binom{2}{3}}\left(e^{-f}\binom{2}{3}e^{-\binom{2}{3}(x-f)}\right)=e^{-3x+2f} & f\le x\le f+y_0 \\ \frac{1}{\binom{2}{4}}\left(e^{-f}e^{-\binom{2}{3}y_0}\binom{2}{4}e^{-\binom{2}{4}(x-f-y_0)}\right)=e^{-6x+5f+3y_0} & f+y_0\le x \end{cases}$$

Let $f_{Z|X}(z;x)$ be the probability density that z is the CU difference in heights of (r,r'), conditioned on that x is the CU difference in heights of (p,p') and p' is the lowest point of coalescence. Notice that:

$$f_{Z|X}(z;x)=\begin{cases} e^{-z} & 0\le x\le f+y_0 \text{ and } 0\le z \\ e^{-\left(z-(x-f-y_0)\right)}=e^{-z+x-f-y_0} & 0\le x-f-y_0\le z \end{cases}$$

We specify three coalescence scenarios by indicator functions $\delta_1,\delta_2,\delta_3$: *i)* δ_1 indicates $0\le x<f$; *ii)* δ_2 indicates $f\le x<f+y_0$; *iii)* δ_3 indicates $f+y_0\le x$.

Note that

$$\mathbb{E}\left[w_G(ab|cd)\right]=\mathbb{E}\left[(\delta_1+\delta_2+\delta_3)w_G(ab|cd)\right]$$
$$\mathbb{E}\left[w_G^2(ab|cd)\right]=\mathbb{E}\left[(\delta_1+\delta_2+\delta_3)w_G^2(ab|cd)\right].$$

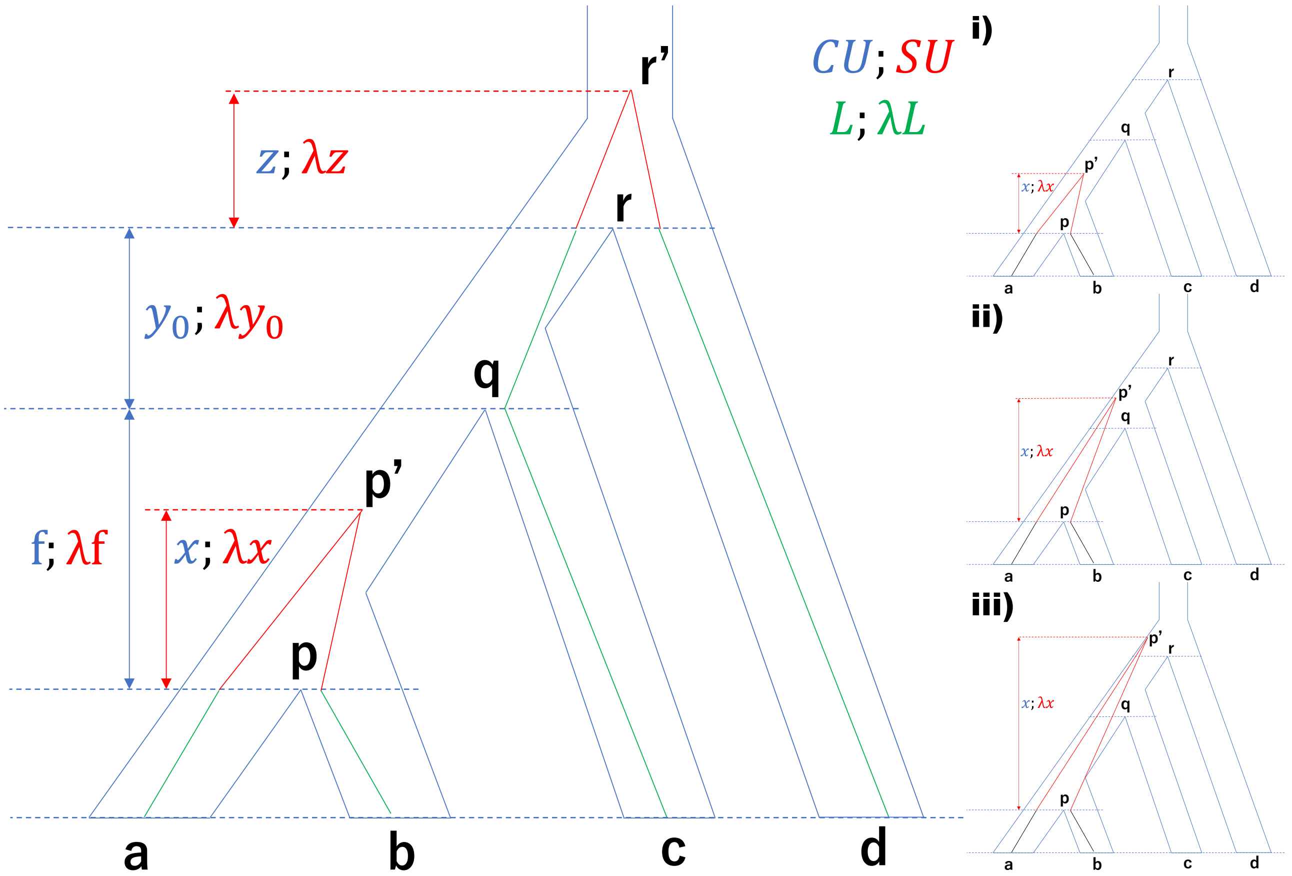

FIG. S23. Illustration of the unbalanced case. Lengths in CU/SU units are denoted in blue/red. Branches in green have a total length $L/\lambda L$ in CU/SU units. The right-hand side shows the position of p' in relation to q and r in various cases.

Similarly, since only scenarios 2 and 3 have deep coalescence events that may lead to gene tree disagreement with the species tree, and by the symmetry of all three topologies under scenarios 2 and 3,

$$\mathbb{E}\big[w_G(ac|bd)\big] = \mathbb{E}\big[(\delta_2+\delta_3)w_G(ab|cd)\big]$$
$$\mathbb{E}\big[w_G^2(ac|bd)\big] = \mathbb{E}\big[(\delta_2+\delta_3)w_G^2(ab|cd)\big]\,.$$

Thus,

$$\mathbb{E}[X_G] = \mathbb{E}\big[w_G(ab|cd)\big] - \mathbb{E}\big[w_G(ac|bd)\big] = \mathbb{E}\big[\delta_1 w_G(ab|cd)\big]\,, \tag{11}$$

and since $w_G(ab|cd)w_G(ac|bd) = 0$,

$$\begin{aligned}\mathrm{Var}[X_G] &= \mathbb{E}[X_G^2] - \mathbb{E}^2[X_G] = \mathbb{E}\big[w_G^2(ab|cd) + w_G^2(ac|bd)\big] - \mathbb{E}^2[X_G] \\ &= \mathbb{E}\big[(\delta_1 + 2\delta_2 + 2\delta_3)w_G^2(ab|cd)\big] - \mathbb{E}^2[X_G]\,.\end{aligned} \tag{12}$$

We next compute both elements of (11) as well as some elements of (12) (others will not be necessary).

- δ_1: When G has topology $ab|cd$, p' must be the lowest point of coalescence. Thus,

$$\begin{aligned}&\mathbb{E}\big[\delta_1 w_G(ab|cd)\big] \\ &= \int_0^f \int_0^{+\infty} e^{-\lambda(2x+2z+L)} f_X(x) f_{Z|X}(z;x)\,dz\,dx \\ &= \int_0^f \int_0^{+\infty} e^{-\lambda(2x+2z+L)} e^{-x} e^{-z}\,dz\,dx \\ &= \frac{e^{-\lambda L}(1-e^{-(1+2\lambda)f})}{(1+2\lambda)^2}\,;\end{aligned}$$

$$\mathbb{E}\left[\delta_1 w_G^2(ab|cd)\right] \le \mathbb{E}\left[\delta_1 w_G(ab|cd)\right] = O(f)\,.$$

- δ_2: When G has topology $ab|cd$, p' must be the lowest point of coalescence. Thus,

$$\begin{aligned}
&\mathbb{E}\left[\delta_2 w_G^2(ab|cd)\right] \\
&= \int_f^{f+y_0}\int_0^{+\infty} e^{-\lambda(4x+4z+2L)} f_X(x) f_{Z|Y}(z;y)\,dz\,dx \\
&= \int_f^{f+y_0}\int_0^{+\infty} e^{-\lambda(4x+4z+2L)} e^{-3x+2f} e^{-z}\,dz\,dx \\
&= \frac{1-e^{-(3+4\lambda)y_0}}{(1+4\lambda)(3+4\lambda)} e^{-(1+4\lambda)f-2\lambda L}\,.
\end{aligned}$$

- δ_3: When G has the topology $ab|cd$, either p' or q' must be the lowest point of coalescence, and by symmetry, the two cases must have the same PDFs. Thus,

$$\begin{aligned}
&\mathbb{E}\left[\delta_3 w_G^2(ab|cd)\right] \\
&= \int_{f+y_0}^{+\infty}\int_{x-f-y_0}^{+\infty} e^{-\lambda(4x+4z+2L)} 2 f_X(x) f_{Z|X}(z;x)\,dz\,dx \\
&= \int_{f+y_0}^{+\infty}\int_{x-f-y_0}^{+\infty} e^{-\lambda(4x+4z+2L)} 2e^{-6x+5f+3y_0} e^{-z+x-f-y_0}\,dz\,dx \\
&= \int_{f+y_0}^{+\infty} e^{-4\lambda(x+x-f-y_0)-2\lambda L} 2e^{-6x+5f+3y_0} \frac{1}{1+4\lambda}\,dx \\
&= \frac{1}{(3+4\lambda)(1+4\lambda)} e^{-(1+4\lambda)f-(3+4\lambda)y_0-2\lambda L}\,.
\end{aligned}$$

Replacing in (11), we get

$$\mathbb{E}[X_G] = \mathbb{E}\left[\delta_1 w_G(ab|cd)\right] = \frac{e^{-\lambda L}(1-e^{-(1+2\lambda)f})}{(1+2\lambda)^2} = \frac{e^{-\lambda L}}{1+2\lambda} f + O(f^2)\,;$$

and replacing in (12), we get

$$\begin{aligned}
\mathrm{Var}[X_G] &= \mathbb{E}\left[(\delta_1+2\delta_2+2\delta_3) w_G^2(ab|cd)\right] - \mathbb{E}^2[X_G] = \mathbb{E}\left[2(\delta_2+\delta_3) w_G^2(ab|cd)\right] + O(f) \\
&= \frac{2e^{-(1+4\lambda)f-2\lambda L}}{(3+4\lambda)(1+4\lambda)} + O(f) = \frac{2e^{-2\lambda L}}{(3+4\lambda)(1+4\lambda)} + O(f)\,,
\end{aligned}$$

from which our assumption of $\mathrm{Var}[X_G] = \Omega(1)$ follows.

Case 2: Balanced tree.

Let p,q, and r be the MRCA nodes of (a,b), (c,d), and (a,d) on rooted species tree S^*, respectively. Let p' and q' be the points of coalescence of leaves a,b and leaves c,d on the rooted gene tree G, respectively. Let x, x_0, y, and y_0 be the CU difference in heights of points (p,p'), (p,r), (q,q'), and (q,r), respectively. Note that $f = x+y$ is CU length of path (p,q). Let $L := l_{S^*}(a,p) + l_{S^*}(b,p) + l_{S^*}(c,q) + l_{S^*}(d,q)$. Notice that $l_G(a,p) + l_G(b,p) + l_G(c,q) + l_G(d,q) = \lambda L$ and $l_G(a,b) + l_G(c,d) = \lambda(2x+2y+L)$.

We specify three coalescence scenarios by indicator functions $\delta_1,\delta_2,\delta_3$: *i)* δ_1 indicates $0 \le x < x_0$; *ii)* δ_2 indicates $x_0 \le x, 0 \le y < y_0$; *iii)* δ_3 indicates $x_0 \le x, y_0 \le y$.

Note that

$$\begin{aligned}
\mathbb{E}\left[w_G(ab|cd)\right] &= \mathbb{E}\left[(\delta_1+\delta_2+\delta_3) w_G(ab|cd)\right] \\
\mathbb{E}\left[w_G^2(ab|cd)\right] &= \mathbb{E}\left[(\delta_1+\delta_2+\delta_3) w_G^2(ab|cd)\right]\,.
\end{aligned}$$

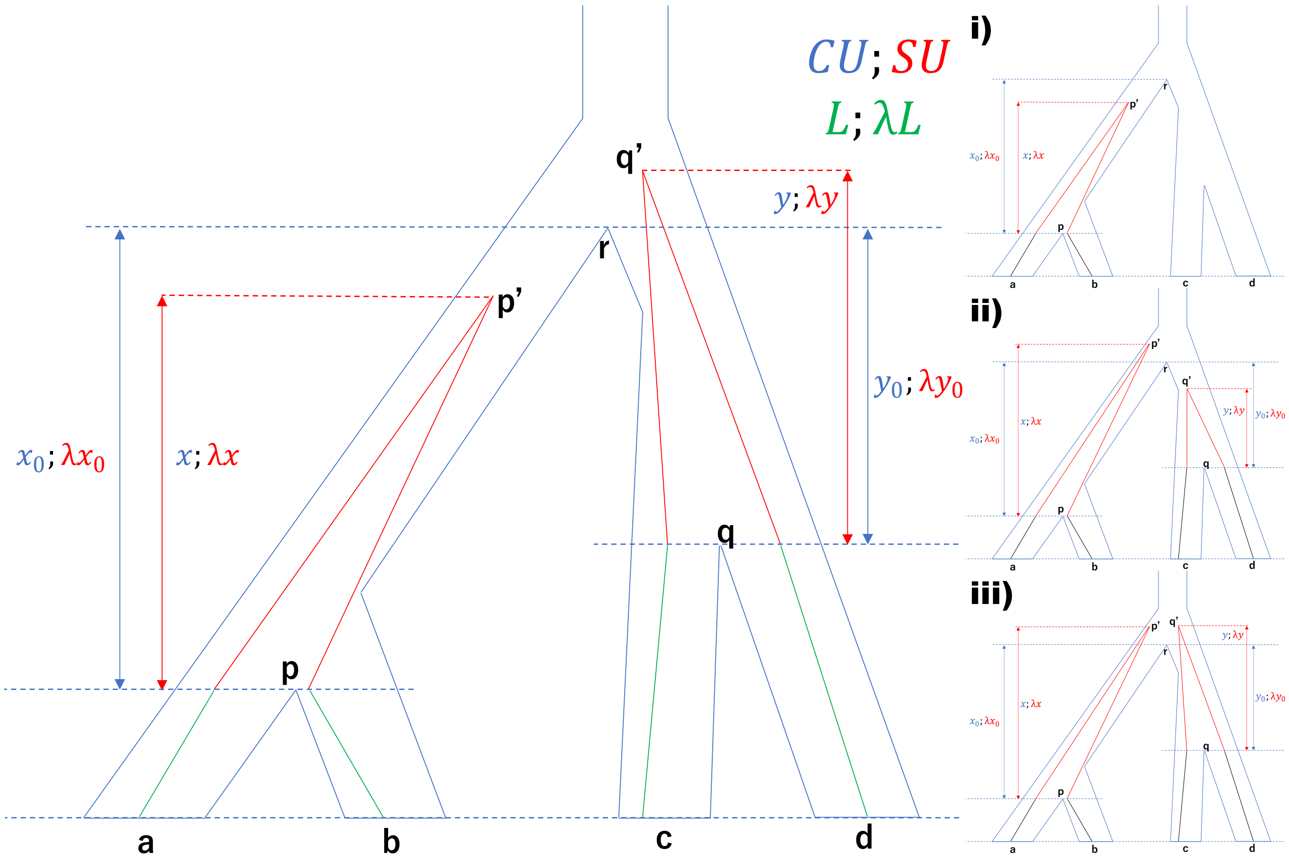

FIG. S24. Illustration of the unbalanced case. Lengths in CU/SU units are denoted in blue/red. Branches in green have a total length $L/\lambda L$ in CU/SU units. The right-hand side shows the position of p' and q' in relation to r in various cases.

Similarly, since only scenarios 3 have deep coalescence events that may lead to gene tree disagreement with the species tree, and by the symmetry of all three topologies under scenarios 3,

$$\mathbb{E}\big[w_G(ac|bd)\big] = \mathbb{E}\big[\delta_3 w_G(ab|cd)\big]$$
$$\mathbb{E}\big[w_G^2(ac|bd)\big] = \mathbb{E}\big[\delta_3 w_G^2(ab|cd)\big]\,.$$

Thus,

$$\mathbb{E}[X_G] = \mathbb{E}\big[w_G(ab|cd)\big] - \mathbb{E}\big[w_G(ac|bd)\big] = \mathbb{E}\big[(\delta_1+\delta_2)w_G(ab|cd)\big]\,; \tag{13}$$

and since $w_G(ab|cd)w_G(ac|bd) = 0$,

$$\begin{aligned}\mathrm{Var}[X_G] &= \mathbb{E}[X_G^2] - \mathbb{E}^2[X_G] = \mathbb{E}\big[w_G^2(ab|cd) + w_G^2(ac|bd)\big] - \mathbb{E}^2[X_G] \\ &= \mathbb{E}\big[(\delta_1+\delta_2+2\delta_3)w_G^2(ab|cd)\big] - \mathbb{E}^2[X_G]\,.\end{aligned} \tag{14}$$

- δ_1: Here,

$$\begin{aligned}\mathbb{E}\big[\delta_1 w_G(ab|cd)\big] &= \int_0^{x_0}\int_0^{+\infty} e^{-\lambda(2x+2y+L)}e^{-x}e^{-y}\,dy\,dx \\ &= \frac{e^{-\lambda L}(1-e^{-(1+2\lambda)x_0})}{(1+2\lambda)^2} = \frac{e^{-\lambda L}x_0}{1+2\lambda} + O(x_0^2) = \frac{e^{-\lambda L}x_0}{1+2\lambda} + O(f^2)\,;\end{aligned}$$

and

$$\mathbb{E}\big[\delta_1 w_G^2(ab|cd)\big] \le \mathbb{E}\big[\delta_1 w_G(ab|cd)\big] = O(f)\,.$$

- δ_2: Here,

$$\mathbb{E}\left[\delta_2 w_G(ab|cd)\right]=\int_{x_0}^{+\infty}\int_0^{y_0} e^{-\lambda(2x+2y+L)}e^{-x}e^{-y}dydx$$
$$=\frac{e^{-\lambda L}(1-e^{-(1+2\lambda)y_0})e^{-(1+2\lambda)x_0}}{(1+2\lambda)^2}=\frac{e^{-\lambda L}y_0}{1+2\lambda}+O(f^2);$$

  and

$$\mathbb{E}\left[\delta_2 w_G^2(ab|cd)\right]\le\mathbb{E}\left[\delta_2 w_G(ab|cd)\right]=O(f).$$

- δ_3: Similar to the unbalanced case, when G has the topology $ab|cd$, either p' or q' must be the lowest point of coalescence, and by symmetry, the two cases must have the same PDFs. Thus,

$$\mathbb{E}\left[\delta_3 w_G^2(ab|cd)\right]=\int_{x_0}^{+\infty}\int_{x-x_0+y_0}^{+\infty} e^{-\lambda(4x+4y+2L)}2e^{-x_0}e^{-y_0}e^{-6x+6x_0}e^{-y+x-x_0+y_0}dydx$$
$$=\int_{x_0}^{+\infty} e^{-4\lambda(x+x-x_0+y_0)-2\lambda L}2e^{-x_0}e^{-y_0}e^{-6x+6x_0}\frac{1}{1+4\lambda}dx$$
$$=\frac{1}{(3+4\lambda)(1+4\lambda)}e^{-(1+4\lambda)(x_0+y_0)-2\lambda L}=\frac{1}{(3+4\lambda)(1+4\lambda)}e^{-(1+4\lambda)f-2\lambda L}.$$

Replacing in (13), we get

$$\mathbb{E}[X_G]=\mathbb{E}\left[(\delta_1+\delta_2)w_G(ab|cd)\right]=\frac{e^{-\lambda L}(x_0+y_0)}{1+2\lambda}+O(f^2)=\frac{e^{-\lambda L}f}{1+2\lambda}+O(f^2);$$

and replacing in (14), we get

$$\mathrm{Var}[X_G]=\mathbb{E}\left[(\delta_1+\delta_2+2\delta_3)w_G^2(ab|cd)\right]-\mathbb{E}^2[X_G]$$
$$=\mathbb{E}\left[2\delta_3 w_G^2(ab|cd)\right]+O(f)$$
$$=\frac{2e^{-(1+4\lambda)f-2\lambda L}}{(3+4\lambda)(1+4\lambda)}+O(f)=\frac{2e^{-2\lambda L}}{(3+4\lambda)(1+4\lambda)}+O(f),$$

from which our assumption of $\mathrm{Var}[X_G]=\Theta_f(1)$ follows.

Thus, in both balanced and unbalanced cases,

$$\frac{\mathbb{E}[X_G]}{\sqrt{\mathrm{Var}[X_G]}}=\frac{\frac{e^{-\lambda L}}{1+2\lambda}f+O(f^2)}{\sqrt{\frac{2e^{-2\lambda L}}{(1+4\lambda)(3+4\lambda)}}+O(f)}=\sqrt{1+\frac{4\lambda+4\lambda^2}{3(1+2\lambda)^2}}\sqrt{\frac{3}{2}}f+O(f^2)$$

□

Proposition 3. *For a true quartet species tree S^* with topology $ab|cd$ and input gene trees $\mathcal{G}$ generated under the variable rate model, let f be the distance between anchors of S^* and L be the total length of all other branches. Assume that for every branch segment I, the variance of its multiplier is bounded above: $Var(\Lambda^I_{S^\dagger})\le\varepsilon^2$ where $\varepsilon^2=\frac{e^{-\lambda L}}{(16+32\lambda)+(6+32\lambda+32\lambda^2)L}\left(\frac{20(\lambda+\lambda^2)}{9(1+2\lambda)^2}\right)^3$. As $f\to 0$, given $k=\Theta(f^{-2})$ gene trees, we have $Var[X_G]=\Theta_f(1)$ and*

$$\frac{\mathbb{E}[X_G]}{\sqrt{Var[X_G]}}\ge\sqrt{\frac{3}{2}}\left(1-\frac{4\lambda^2}{(1+4\lambda)^2}\right)^{-\frac{1}{2}}f+O(f^2).$$

Proof. We follow the same logic in proof of Proposition 2.

Case 1: Unbalanced trees. Let $P(x)$ be functions to random variables denoting SU difference in heights of points (p,p') where p' is x CU distance above p; let $R(z)$ be functions to random variables denoting SU difference in heights of points (r,r') where r' is z CU distance above r. Note that $P(f+y_0)+R(z)=P(f+y_0+z)$ where $P(f+y_0)$ denote the SU length of (p,r). Let random variable $\Lambda := \big(l_{S^\dagger}(a,p)+l_{S^\dagger}(b,p)+l_{S^\dagger}(c,r)+l_{S^\dagger}(d,r)\big)$ be the total SU terminal branch lengths and the constant value L be the CU distance corresponding to Λ.

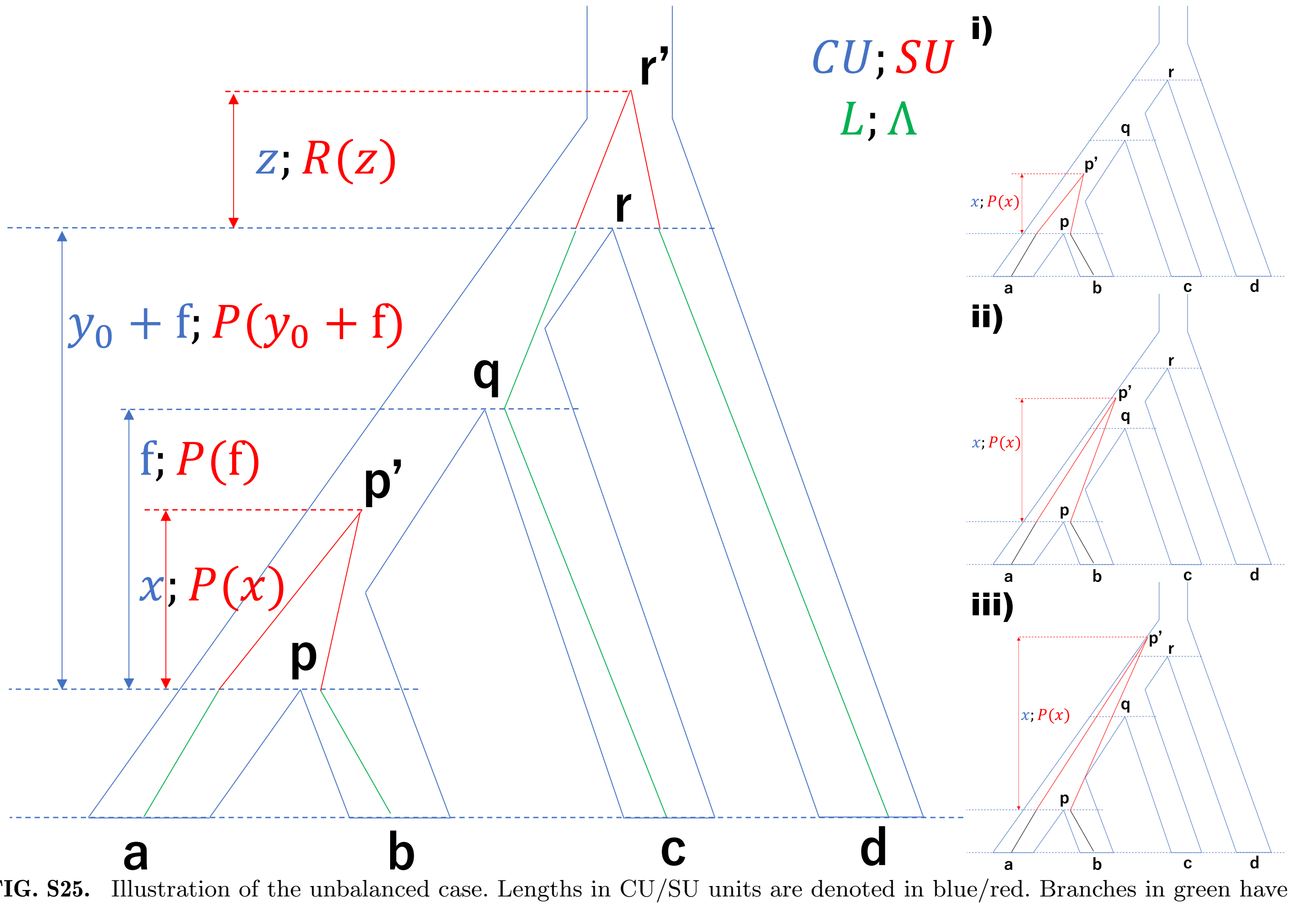

FIG. S25. Illustration of the unbalanced case. Lengths in CU/SU units are denoted in blue/red. Branches in green have a total length L/Λ in CU/SU units. The right-hand side shows the position of p' in relation to q and r in various cases.

- δ_1: When G has topology $ab|cd$, p' must be the lowest point of coalescence. Thus,

$$\begin{aligned}&\mathbb{E}\big[\delta_1 w_G(ab|cd)\big]\\ =&\mathbb{E}\Big[\int_0^f\int_0^{+\infty} e^{-2P(x)-2R(z)-\Lambda} f_X(x) f_{Z|X}(z;x)dzdx\Big]\\ =&\mathbb{E}\Big[\int_0^f\int_0^{+\infty} e^{-2P(x)-2R(z)-\Lambda} e^{-x}e^{-z}dzdx\Big]\\ =&\mathbb{E}\Big[\int_0^f\int_0^{+\infty} e^{-2P(x)-2R(z)-\Lambda-x-z}dzdx\Big];\end{aligned}$$

and

$$\mathbb{E}\big[\delta_1 w_G^2(ab|cd)\big] \le \mathbb{E}\big[\delta_1 w_G(ab|cd)\big] = O(f).$$

- δ_2: When G has topology $ab|cd$, p' must be the lowest point of coalescence. Thus,

$$\begin{aligned}
&\mathbb{E}\big[\delta_2 w_G^2(ab|cd)\big]\\
=&\mathbb{E}\Big[\int_f^{f+y_0}\int_0^{+\infty} e^{-4P(x)-4R(z)-2\Lambda} f_X(x) f_{Z|Y}(z;y)\,dzdx\Big]\\
=&\mathbb{E}\Big[\int_f^{f+y_0}\int_0^{+\infty} e^{-4P(x)-4R(z)-2\Lambda} e^{-3x+2f} e^{-z}\,dzdx\Big]\\
=&\int_f^{f+y_0}\int_0^{+\infty} \mathbb{E}\big[e^{-4P(x)-4R(z)-2\Lambda}\big] e^{-3x-z+2f}\,dzdx\,.
\end{aligned}$$

- δ_3: When G has the topology $ab|cd$, either p' or q' must be the lowest point of coalescence, and by symmetry, the two cases must have the same PDFs. Thus,

$$\begin{aligned}
&\mathbb{E}\big[\delta_3 w_G^2(ab|cd)\big]\\
=&\mathbb{E}\Big[\int_{f+y_0}^{+\infty}\int_{x-f-y_0}^{+\infty} e^{-4P(x)-4R(z)-2\Lambda} 2 f_X(x) f_{Z|X}(z;x)\,dzdx\Big]\\
=&\mathbb{E}\Big[\int_{f+y_0}^{+\infty}\int_{x-f-y_0}^{+\infty} e^{-4P(x)-4R(z)-2\Lambda} 2e^{-6x+5f+3y_0} e^{-z+x-f-y_0}\,dzdx\Big]\\
=&\int_{f+y_0}^{+\infty}\int_{x-f-y_0}^{+\infty} \mathbb{E}\big[e^{-4P(x)-4R(z)-2\Lambda}\big] 2e^{-5x-z+4f+2y_0}\,dzdx\,.
\end{aligned}$$

Replacing in (11), by Jensen's inequality, we get

$$\begin{aligned}
\mathbb{E}[X_G]=&\mathbb{E}\big[\delta_1 w_G(ab|cd)\big]=\mathbb{E}\Big[\int_0^f\int_0^{+\infty} e^{-2P(x)-2R(z)-\Lambda-x-z}\,dzdx\Big]\\
\geq&\int_0^f\int_0^{+\infty} e^{\mathbb{E}\left[-2P(x)-2R(z)-\Lambda-x-z\right]}\,dzdx\\
=&\int_0^f\int_0^{+\infty} e^{-2\lambda x-2\lambda z-\lambda L-x-z}\,dzdx\\
=&\frac{e^{-\lambda L}(1-e^{-(1+2\lambda)f})}{(1+2\lambda)^2}=\frac{e^{-\lambda L}}{1+2\lambda}f+O(f^2)\,.
\end{aligned}$$

And replacing in (12), we get

$$\begin{aligned}
\mathrm{Var}[X_G]=&\mathbb{E}\big[(\delta_1+2\delta_2+2\delta_3)w_G^2(ab|cd)\big]-\mathbb{E}^2[X_G]=\mathbb{E}\big[2(\delta_2+\delta_3)w_G^2(ab|cd)\big]+O(f)\\
=&\int_f^{f+y_0}\int_0^{+\infty}\mathbb{E}\big[e^{-4P(x)-4R(z)-2\Lambda}\big]2e^{-3x-z+2f}\,dzdx\\
&+\int_{f+y_0}^{+\infty}\int_{x-f-y_0}^{+\infty}\mathbb{E}\big[e^{-4P(x)-4R(z)-2\Lambda}\big]4e^{-5x-z+2f+2y_0}\,dzdx+O(f)\,,
\end{aligned}$$

from which our assumption of $\mathrm{Var}[X_{G^*}]=\Theta_f(1)$ follows.

Let $F_P(u;x)$, $F_R(v;z)$, and $F_\Lambda(w)$ be the CDF of $P(x)$, $R(z)$, and Λ respectively; let $F_{PR\Lambda}(u,v,w;x,z)$ and $F_{PR\Lambda}(u,v,w;x,z)$ be the joint CDF and the joint PDF. Let $F_P^{-1}(t;x)$, $F_R^{-1}(t;z)$, and $F_\Lambda^{-1}(t)$ be the inverse function of CDF of $P(x)$, $R(z)$, and Λ respectively.

Then,

$$
\begin{aligned}
&\mathbb{E}\Big[e^{-2\big(2P(x)+2R(z)+\Lambda\big)}\Big]\\
=&\int_0^{+\infty}\int_0^{+\infty}\int_0^{+\infty} e^{-2(2u+2v+w)} F_{PR\Lambda}(u,v,w;x,z)dwdvdu\\
=&\int_0^{+\infty}\int_0^{+\infty}\int_0^{+\infty} e^{-2(2u+2v+w)} \frac{\partial^3 F_{PR\Lambda}}{\partial u\partial v\partial w}dwdvdu\\
=&\int_0^{+\infty}\int_0^{+\infty}\Big(e^{-2(2u+2v+w)}\frac{\partial^2 F_{PR\Lambda}}{\partial u\partial v}\Big|_{w=0}^{+\infty}\\
&-\int_0^{+\infty}(-2)e^{-2(2u+2v+w)}\frac{\partial^2 F_{PR\Lambda}}{\partial u\partial v}dw\Big)dvdu\\
=&\int_0^{+\infty}\int_0^{+\infty}\int_0^{+\infty} 2e^{-2(2u+2v+w)}\frac{\partial^2 F_{PR\Lambda}}{\partial u\partial v}dvdudw\\
=&\int_0^{+\infty}\int_0^{+\infty}\Big(2e^{-2(2u+2v+w)}\frac{\partial F_{PR\Lambda}}{\partial u}\Big|_{v=0}^{+\infty}-\int_0^{+\infty}(-8)e^{-2(2u+2v+w)}\frac{\partial F_{PR\Lambda}}{\partial u}dv\Big)dudw\\
=&\int_0^{+\infty}\int_0^{+\infty}\int_0^{+\infty} 8e^{-2(2u+2v+w)}\frac{\partial F_{PR\Lambda}}{\partial u}dudwdv\\
=&\int_0^{+\infty}\int_0^{+\infty}\Big(8e^{-2(2u+2v+w)}F_{PR\Lambda}(u,v,w;x,z)\Big|_{u=0}^{+\infty}\\
&-\int_0^{+\infty}(-32)e^{-2(2u+2v+w)}F_{PR\Lambda}(u,v,w;x,z)du\Big)dwdv\\
=&\int_0^{+\infty}\int_0^{+\infty}\int_0^{+\infty} 32e^{-2(2u+2v+w)}F_{PR\Lambda}(u,v,w;x,z)dwdvdu\\
\le&\int_0^{+\infty}\int_0^{+\infty}\int_0^{+\infty} 32e^{-2(2u+2v+w)}\min\{F_P(u;x),F_R(v;z),F_\Lambda(w)\}dwdvdu\\
=&\int_0^{+\infty}\int_0^{+\infty}\int_0^{+\infty} 32e^{-2(2u+2v+w)}\Big(\int_0^1 1_{t\le F_P(u;x)}1_{t\le F_R(v;z)}1_{t\le F_\Lambda(w)}dt\Big)dwdvdu\\
=&\int_0^1\int_0^{+\infty}\int_0^{+\infty}\int_0^{+\infty} 32e^{-2(2u+2v+w)}1_{u\ge F_P^{-1}(t;x)}1_{v\ge F_R^{-1}(t;z)}1_{w\ge F_\Lambda^{-1}(t)}dwdvdudt\\
=&\int_0^1\int_{F_P^{-1}(t;x)}^{+\infty}\int_{F_R^{-1}(t;z)}^{+\infty}\int_{F_\Lambda^{-1}(t)}^{+\infty} 32e^{-2(2u+2v+w)}dwdvdudt\\
=&\int_0^1 e^{-2\big(2F_P^{-1}(t;x)+2F_R^{-1}(t;z)+F_\Lambda^{-1}(t)\big)}dt.
\end{aligned}
$$

Thus, for any $0<t_0<1$,

$$
\begin{aligned}
&\mathbb{E}\Big[e^{-2\big(2P(x)+2R(z)+\Lambda\big)}\Big]\\
\le&\int_0^1 e^{-2\big(2F_P^{-1}(t;x)+2F_R^{-1}(t;z)+F_\Lambda^{-1}(t)\big)}dt\\
\le&\int_0^{t_0}\overbrace{e^{-2\big(2F_P^{-1}(0;x)+2F_R^{-1}(0;z)+F_\Lambda^{-1}(0)\big)}}^{1}dt+\int_{t_0}^1 e^{-2\big(2F_P^{-1}(t_0;x)+2F_R^{-1}(t_0;z)+F_\Lambda^{-1}(t_0)\big)}dt\\
\le& t_0+e^{-2\big(2F_P^{-1}(t_0;x)+2F_R^{-1}(t_0;z)+F_\Lambda^{-1}(t_0)\big)}.
\end{aligned}
$$

By Chebyshev's inequality (using $t_0^{-\frac{1}{2}}$ as the constant), $F_P^{-1}(t_0;x)\ge(\lambda-\frac{\varepsilon}{\sqrt{t_0}})x$, $F_R^{-1}(t_0;z)\ge(\lambda-\frac{\varepsilon}{\sqrt{t_0}})y$, and $F_\Lambda^{-1}(t_0)\ge(\lambda-\frac{\varepsilon}{\sqrt{t_0}})L$. Thus,

$$
\mathbb{E}\Big[e^{-2\big(2P(x)+2R(z)+\Lambda\big)}\Big]\le t_0+e^{(-\lambda+\frac{\varepsilon}{\sqrt{t_0}})(4x+4z+2L)}.
$$

Thus,

$$
\begin{aligned}
\mathrm{Var}[X_{G^*}] \leq & \int_f^{f+y_0} \int_0^{+\infty} \Big(t_0 + e^{(-\lambda+\frac{\varepsilon}{\sqrt{t_0}})(4x+4z+2L)}\Big) 2e^{-3x-z+2f} dzdx \\
& + \int_{f+y_0}^{+\infty} \int_{x-f-y_0}^{+\infty} \Big(t_0 + e^{(-\lambda+\frac{\varepsilon}{\sqrt{t_0}})(4x+4z+2L)}\Big) 4e^{-5x-z+2f+2y_0} dzdx + O(f) \\
= & \int_f^{f+y_0} \Big(2t_0 e^{-3x+2f} + \frac{2}{1+4\lambda-\frac{4\varepsilon}{\sqrt{t_0}}} e^{(-\lambda+\frac{\varepsilon}{\sqrt{t_0}})(4x+2L)-3x+2f}\Big) dx \\
& + \int_{f+y_0}^{+\infty} \Big(4t_0 e^{-6x+3f+3y_0} + \frac{4}{1+4\lambda-\frac{4\varepsilon}{\sqrt{t_0}}} e^{(-\lambda+\frac{\varepsilon}{\sqrt{t_0}})(8x-4f-4y_0+2L)-6x+3f+3y_0}\Big) dx + O(f) \\
= & \frac{2}{3} t_0 (e^{-f} - e^{-f-3y_0}) + \frac{2}{(1+4\lambda-\frac{4\varepsilon}{\sqrt{t_0}})(3+4\lambda-\frac{4\varepsilon}{\sqrt{t_0}})} \big(e^{(-\lambda+\frac{\varepsilon}{\sqrt{t_0}})(4f+2L)-f} - e^{(-\lambda+\frac{\varepsilon}{\sqrt{t_0}})(4f+4y_0+2L)-f-3y_0}\big) \\
& + \frac{4}{6} t_0 e^{-3f-3y_0} + \frac{4}{(1+4\lambda-\frac{4\varepsilon}{\sqrt{t_0}})(6+8\lambda-\frac{8\varepsilon}{\sqrt{t_0}})} e^{(-\lambda+\frac{\varepsilon}{\sqrt{t_0}})(4f+4y_0+2L)-3f-3y_0} + O(f) \\
= & \frac{2}{3} t_0 + \frac{2e^{-2L(\lambda-\frac{\varepsilon}{\sqrt{t_0}})}}{(1+4\lambda-\frac{4\varepsilon}{\sqrt{t_0}})(3+4\lambda-\frac{4\varepsilon}{\sqrt{t_0}})} + O(f).
\end{aligned}
$$

Case 2: Balanced tree. Let $P(x)$ be functions to random variables denoting SU difference in heights of points (p,p') where p' is x CU distance above p; let $Q(y)$ be functions to random variables denoting SU difference in heights of points (q,q') where q' is y CU distance above q. Note that $P(x_0+z)-P(x_0) = Q(y_0+z)-Q(y_0)$ where $P(x_0)$ and $Q(y_0)$ denote the SU length of (p,r) and (q,r), respectively. Let random variable $\Lambda := \big(l_{S^\dagger}(a,p)+l_{S^\dagger}(b,p)+l_{S^\dagger}(c,q)+l_{S^\dagger}(d,q)\big)$ be the total SU terminal branch lengths and the constant value L be the CU distance corresponding to Λ.

- δ_1: Here,

  $$\mathbb{E}\big[\delta_1 w_G(ab|cd)\big] = \mathbb{E}\Big[\int_0^{x_0} \int_0^{+\infty} e^{-2P(x)-2Q(y)-\Lambda} e^{-x} e^{-y} dydx\Big];$$

  and

  $$\mathbb{E}\big[\delta_1 w_G^2(ab|cd)\big] \leq \mathbb{E}\big[\delta_1 w_G(ab|cd)\big] = O(f).$$

- δ_2: Here,

  $$\mathbb{E}\big[\delta_2 w_G(ab|cd)\big] = \mathbb{E}\Big[\int_{x_0}^{+\infty} \int_0^{y_0} e^{-2P(x)-2Q(y)-\Lambda} e^{-x} e^{-y} dydx\Big];$$

  and

  $$\mathbb{E}\big[\delta_2 w_G^2(ab|cd)\big] \leq \mathbb{E}\big[\delta_2 w_G(ab|cd)\big] = O(f).$$

- δ_3: Similar to the unbalanced case, when G has the topology $ab|cd$, either p' or q' must be the lowest point of coalescence, and by symmetry, the two cases must have the same PDFs. Thus,

  $$
  \begin{aligned}
  \mathbb{E}\big[\delta_3 w_G^2(ab|cd)\big] = & \mathbb{E}\Big[\int_{x_0}^{+\infty} \int_{x-x_0+y_0}^{+\infty} e^{-4P(x)-4Q(y)-2\Lambda} 2e^{-x_0} e^{-y_0} e^{-6x+6x_0} e^{-y+x-x_0+y_0} dydx\Big] \\
  = & \int_{x_0}^{+\infty} \int_{x-x_0+y_0}^{+\infty} \mathbb{E}\Big[e^{-4P(x)-4Q(y)-2\Lambda}\Big] 2e^{-5x-y+4x_0} dydx.
  \end{aligned}
  $$

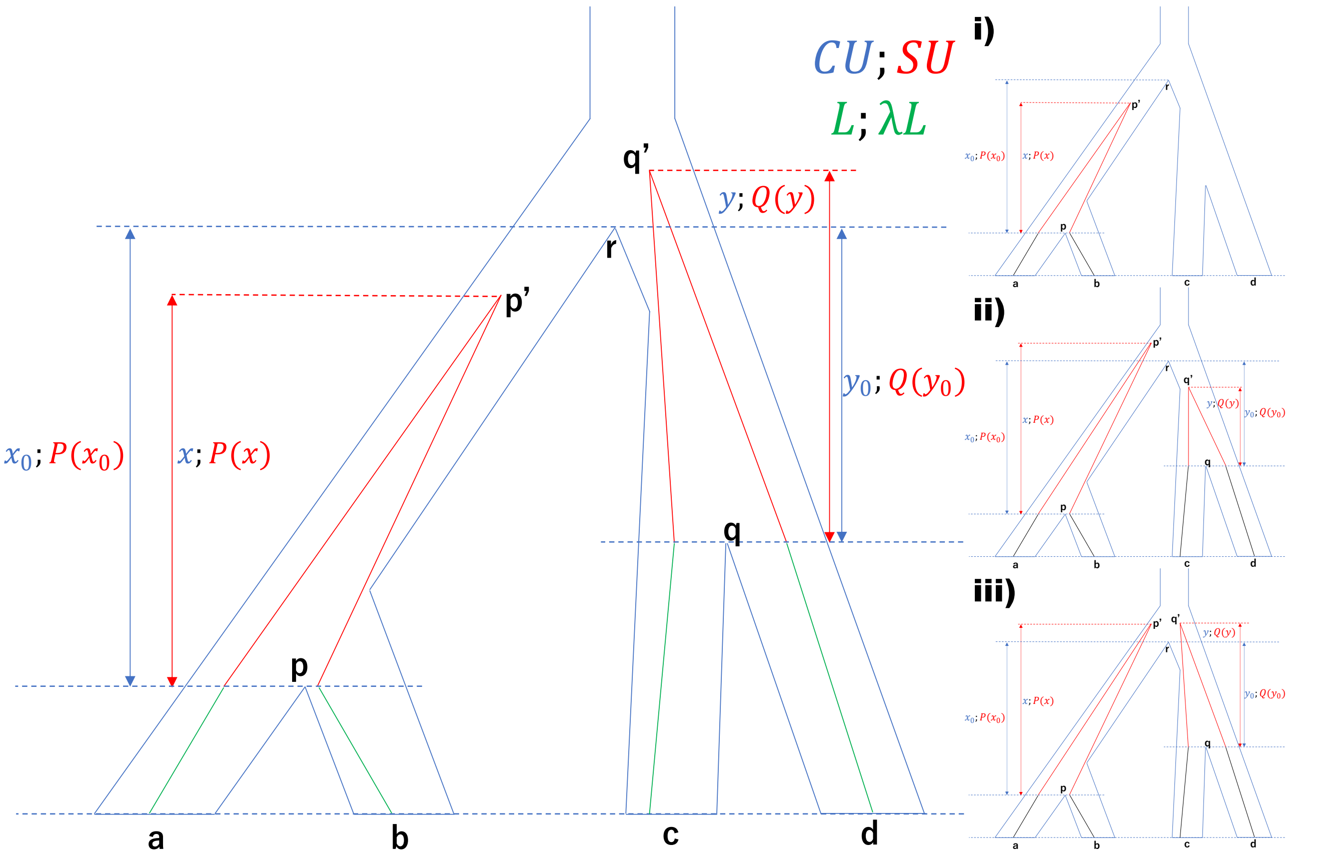

FIG. S26. Illustration of the unbalanced case. Lengths in CU/SU units are denoted in blue/red. Branches in green have a total length L/Λ in CU/SU units. The right-hand side shows the position of p' and q' in relation to r in various cases.

Replacing in (13), we get

$$\begin{aligned}
\mathbb{E}[X_G] &= \mathbb{E}\left[(\delta_1+\delta_2)w_G(ab|cd)\right]\\
&= \mathbb{E}\Big[\int_0^{x_0}\int_0^{+\infty} e^{-2P(x)-2Q(y)-\Lambda}e^{-x}e^{-y}dydx + \int_{x_0}^{+\infty}\int_0^{y_0} e^{-2P(x)-2Q(y)-\Lambda}e^{-x}e^{-y}dydx\Big]\\
&\geq \int_0^{x_0}\int_0^{+\infty} e^{-2\lambda x-2\lambda y-\lambda L}e^{-x}e^{-y}dydx + \int_{x_0}^{+\infty}\int_0^{y_0} e^{-2\lambda x-2\lambda y-\lambda L}e^{-x}e^{-y}dydx\\
&= \frac{(x_0+y_0)e^{-\lambda L}}{1+2\lambda} + O(f^2) = \frac{fe^{-\lambda L}}{1+2\lambda} + O(f^2);
\end{aligned}$$

and replacing in (14), for any $0<t_0<1$,

$$\begin{aligned}
\operatorname{Var}[X_G] &= \mathbb{E}\big[(\delta_1+\delta_2+2\delta_3)w_G^2(ab|cd)\big]-\mathbb{E}^2[X_G]\\
&= \mathbb{E}\big[2\delta_3 w_G^2(ab|cd)\big]+O(f)\\
&= \int_{x_0}^{+\infty}\int_{x-x_0+y_0}^{+\infty}\mathbb{E}\Big[e^{-4P(x)-4Q(y)-2\Lambda}\Big]4e^{-5x-y+4x_0}dydx\\
&\le \int_{x_0}^{+\infty}\int_{x-x_0+y_0}^{+\infty}\Big(t_0+e^{(-\lambda+\frac{\varepsilon}{\sqrt{t_0}})(4x+4y+2L)}\Big)4e^{-5x-y+4x_0}dydx+O(f)\\
&= \int_{x_0}^{+\infty}\Big(4e^{-6x-y_0+5x_0}t_0+\frac{4}{1+4\lambda-\frac{4\varepsilon}{\sqrt{t_0}}}e^{-6x-y_0+5x_0+(-\lambda+\frac{\varepsilon}{\sqrt{t_0}})(8x-4x_0+4y_0+2L)}\Big)dx+O(f)\\
&= \frac{4}{6}e^{-x_0-y_0}t_0+\frac{4}{(1+4\lambda-\frac{4\varepsilon}{\sqrt{t_0}})(6+8\lambda-\frac{8\varepsilon}{\sqrt{t_0}})}e^{-x_0-y_0+(-\lambda+\frac{\varepsilon}{\sqrt{t_0}})(4x_0+4y_0+2L)}+O(f)\\
&= \frac{2}{3}t_0+\frac{2e^{-2L(\lambda-\frac{\varepsilon}{\sqrt{t_0}})}}{(1+4\lambda-\frac{4\varepsilon}{\sqrt{t_0}})(3+4\lambda-\frac{4\varepsilon}{\sqrt{t_0}})}+O(f),
\end{aligned}$$

from which our assumption of $\operatorname{Var}[X_G]=\Theta_f(1)$ follows.

Thus, for both balanced and unbalanced trees, the variance is bounded the by same expression, and thus in both cases,

$$\begin{aligned}
\operatorname{Var}[X_{G^*}] &\le \frac{2}{3}t_0+2\frac{\frac{e^{-2\lambda L}}{(1+4\lambda)(3+4\lambda)}}{(1-\frac{4\varepsilon}{(1+4\lambda)\sqrt{t_0}})(1-\frac{4\varepsilon}{(3+4\lambda)\sqrt{t_0}})e^{-\frac{2\varepsilon L}{\sqrt{t_0}}}}+O(f)\\
&\le \frac{2}{3}t_0+2\frac{\frac{e^{-2\lambda L}}{(1+4\lambda)(3+4\lambda)}}{(1-\frac{4\varepsilon}{(1+4\lambda)\sqrt{t_0}})(1-\frac{4\varepsilon}{(3+4\lambda)\sqrt{t_0}})(1-\frac{2\varepsilon L}{\sqrt{t_0}})}+O(f)\\
&\le \frac{2}{3}t_0+2\frac{\frac{e^{-2\lambda L}}{(1+4\lambda)(3+4\lambda)}}{(1-\frac{4\varepsilon}{(1+4\lambda)\sqrt{t_0}}-\frac{4\varepsilon}{(3+4\lambda)\sqrt{t_0}}-\frac{2\varepsilon L}{\sqrt{t_0}})}+O(f)\\
&= \frac{2}{3}t_0+\frac{2e^{-2\lambda L}}{(3+16\lambda+16\lambda^2)-\frac{\varepsilon}{\sqrt{t_0}}\big((16+32\lambda)+(6+32\lambda+32\lambda^2)L\big)}+O(f).
\end{aligned}$$

Now, let $C:=(16+32\lambda)+(6+32\lambda+32\lambda^2)L, t_0=\big(\frac{C^{\frac{1}{3}}\varepsilon^{\frac{1}{3}}}{(3+16\lambda+16\lambda^2)e^{\frac{2}{3}\lambda L}}\big)^2$, we get

$$\begin{aligned}
\operatorname{Var}[X_{G^*}] &\le \frac{2e^{-2\lambda L}}{3(3+16\lambda+16\lambda^2)^2}\Big((\varepsilon e^{\lambda L}C)^{\frac{2}{3}}+\frac{9+48\lambda+48\lambda^2}{1-(\varepsilon e^{\lambda L}C)^{\frac{2}{3}}}\Big)+O(f)\\
&= \frac{2e^{-2\lambda L}}{3(3+16\lambda+16\lambda^2)}\Big(\frac{(\varepsilon e^{\lambda L}C)^{\frac{2}{3}}}{3+16\lambda+16\lambda^2}+3+\frac{3(\varepsilon e^{\lambda L}C)^{\frac{2}{3}}}{1-(\varepsilon e^{\lambda L}C)^{\frac{2}{3}}}\Big)+O(f).
\end{aligned}$$

Now, recalling that $\varepsilon=\frac{e^{-\lambda L}}{C}\Big(\frac{20(\lambda+\lambda^2)}{9(1+2\lambda)^2}\Big)^{\frac{3}{2}}$,

$$
\begin{aligned}
\mathrm{Var}[X_{G^*}] \le & \frac{2}{3(3+16\lambda+16\lambda^2)(1+2\lambda)^2} \\
& \Big(\frac{\frac{20}{9}(\lambda+\lambda^2)}{3+16\lambda+16\lambda^2}+3+\frac{3(\frac{20}{9})(\lambda+\lambda^2)}{1-\frac{\frac{20}{9}(\lambda+\lambda^2)}{1+4\lambda+4\lambda^2}}\Big)+O(f) \\
\le & \frac{2}{3(3+16\lambda+16\lambda^2)(1+2\lambda)^2}\Big(\frac{20}{27}\lambda+3+\frac{\frac{20}{3}(\lambda+\lambda^2)}{1-\frac{5}{9}}\Big)+O(f) \\
= & \frac{2}{3(3+16\lambda+16\lambda^2)(1+2\lambda)^2}\Big(\frac{20}{27}\lambda+3+15(\lambda+\lambda^2)\Big)+O(f) \\
< & \frac{2}{3(1+2\lambda)^2}\Big(\frac{3+16\lambda+15\lambda^2}{3+16\lambda+16\lambda^2}\Big)+O(f).
\end{aligned}
$$

□

THEOREM 2. *Under the conditions of Proposition 2 or Proposition 3,*

$$P\Big(\sum_{G\in\mathcal{G}} w_G(ab|cd)\le\sum_{G\in\mathcal{G}} w_G(ac|bd)\Big)\le P\Big(\sum_{G\in\mathcal{G}}\delta_G(ab|cd)\le\sum_{G\in\mathcal{G}}\delta_G(ac|bd)\Big).$$

Proof. We start with proving this theorem under the conditions of Proposition 2. Recall $X_G := w_G(ab|cd)-w_G(ac|bd)$ and $Y_G := \delta_G(ab|cd)-\delta_G(ac|bd)$, and let $\bar{X_\mathcal{G}}=\frac{1}{k}\sum_{G\in\mathcal{G}}X_G$ and $\bar{Y_\mathcal{G}}=\frac{1}{k}\sum_{G\in\mathcal{G}}Y_G$. Recall also that under Proposition 2, proved below, under conditions of Theorem 2, we have $\mathrm{Var}[X_G]=\Omega(1)$ and

$$\frac{\mathbb{E}[X_G]}{\sqrt{\mathrm{Var}[X_G]}}=-\sqrt{\frac{3+16\lambda+16\lambda^2}{3+16\lambda+15\lambda^2}}\sqrt{\frac{3}{2}}f+O(f^2). \tag{15}$$

Similarly, we can compute the ratio of mean and variance for Y (corresponding to unweighted ASTRAL):

$$\mathbb{E}[Y_G]:=\mathbb{E}\big[\delta_G(ab|cd)-\delta_G(ac|bd)\big]=1-e^{-f}=f+O(f^2)$$

$$\mathrm{Var}[Y_G]:=\mathrm{Var}\big[\delta_G(ab|cd)-\delta_G(ac|bd)\big]=\frac{5}{3}e^{-f}-e^{-2f}=\frac{2}{3}+O(f)$$

and thus,

$$\frac{\mathbb{E}[Y_G]}{\sqrt{\mathrm{Var}[Y_G]}}=\sqrt{\frac{3}{2}}f+O(f^2). \tag{16}$$

Given Proposition 2, we can use Berry–Esseen theorem to derive

$$
\begin{aligned}
\mathrm{P}\big(\bar{X_\mathcal{G}}\le 0\big)=&\mathrm{P}\big(\frac{\sqrt{k}}{\sqrt{\mathrm{Var}[X_G]}}(\bar{X_\mathcal{G}}-\mathbb{E}[X_G])\le-\frac{\sqrt{k}}{\sqrt{\mathrm{Var}[X_G]}}\mathbb{E}[X_G]\big)= \\
&\Phi(-\sqrt{k}\frac{\mathbb{E}[X_G]}{\sqrt{\mathrm{Var}[X_G]}})+O(\frac{1}{\sqrt{k}}),
\end{aligned}
$$

where Φ denotes CDF of the standard Normal distribution. Since $k=\Theta(f^{-2})$,

$$\mathrm{P}\big(\bar{X_\mathcal{G}}\le 0\big)=\Phi(-\sqrt{k}\frac{\mathbb{E}[X_G]}{\sqrt{\mathrm{Var}[X_G]}})+O(f) \tag{17}$$

and

$$\mathrm{P}\big(\bar{Y_\mathcal{G}}\le 0\big)=\Phi(-\sqrt{k}\frac{\mathbb{E}[Y_G]}{\sqrt{\mathrm{Var}[Y_G]}})+O(f), \tag{18}$$

Combining equations (17) and (18) with (15) and (16), we get

$$\mathrm{P}\big(\sum_{G\in\mathcal{G}} w_G(ab|cd) \leq \sum_{G\in\mathcal{G}} w_G(ac|bd)\big) = \Phi(-\sqrt{\frac{3+16\lambda+16\lambda^2}{3+16\lambda+15\lambda^2}}\sqrt{\frac{3}{2}} f\sqrt{k}) + O(f)$$

and

$$\mathrm{P}\big(\sum_{G\in\mathcal{G}} \delta_G(ab|cd) \leq \sum_{G\in\mathcal{G}} \delta_G(ac|bd)\big) = \Phi(-\sqrt{\frac{3}{2}} f\sqrt{k}) + O(f)\,.$$

As $f\to 0$, the interval $(-\sqrt{1+\frac{4\lambda+4\lambda^2}{3(1+2\lambda)^2}}\sqrt{\frac{3}{2}}f\sqrt{k}, -\sqrt{\frac{3}{2}}f\sqrt{k})$ does not shrink because $\Theta(f\sqrt{k})=\Theta(1)$. Thus, we have

$$\Phi(-\sqrt{\frac{3}{2}} f\sqrt{k}) - \Phi(-\sqrt{1+\frac{4\lambda+4\lambda^2}{3(1+2\lambda)^2}}\sqrt{\frac{3}{2}} f\sqrt{k}) = \Theta(1)$$

ensuring that

$$\mathrm{P}\big(\sum_{G\in\mathcal{G}} w_G(ab|cd) \leq \sum_{G\in\mathcal{G}} w_G(ac|bd)\big) \leq \mathrm{P}\big(\sum_{G\in\mathcal{G}} \delta_G(ab|cd) \leq \sum_{G\in\mathcal{G}} \delta_G(ac|bd)\big)\,.$$

The proof under Proposition 3 is similar. Recall that under Proposition 3, $\mathrm{Var}[X_{G^*}] = \Theta_f(1)$ and

$$\frac{\mathbb{E}[X_{G^*}]}{\sqrt{\mathrm{Var}[X_{G^*}]}} \geq \sqrt{\frac{3}{2}}\Big(1-\frac{4\lambda^2}{(1+4\lambda)^2}\Big)^{-\frac{1}{2}} f + O(f^2)\,. \tag{19}$$

Given this result, the rest of the proof is similar to the proof under the conditions of Proposition 2, culminating in

$$\mathrm{P}\big(\sum_{G^*\in\mathcal{G}} w_{G^*}(ab|cd) \leq \sum_{G^*\in\mathcal{G}} w_{G^*}(ac|bd)\big) \leq \Phi\left(-\Big(1-\frac{4\lambda^2}{(1+4\lambda)^2}\Big)^{-\frac{1}{2}}\sqrt{\frac{3}{2}} f\sqrt{k}\right) + O(f)\,.$$

□

Placement-based Algorithm

In this section, for a node v in tree G, we let $\mathcal{L}_v$ denote the set of leaves under v.

Proof of Theorem 3

THEOREM 3. *Let S be a species tree, i be a species not in $\mathcal{L}_S$, $\mathcal{S}$ be the set of possible species tree topologies by placing i onto S, and S' be the output of Algorithm S1. Then, $W(S',\mathcal{G}) = \max_{\hat{S}\in\mathcal{S}} W(\hat{S},\mathcal{G})$.*

Proof. We start with two propositions, proved below.

PROPOSITION 5. *After each call to* `ColorLeafSet`$(\mathcal{L}^*,X,T,\mathcal{G},W)$ *with a $T\neq\emptyset$, $W[T] = \sum_{G\in\mathcal{G}} W(T,G)$.*

PROPOSITION 6. *Before calling* `OptimalTreeDP` *in line 6 of Algorithm S1, lookup table W contains all tripartitions corresponding to internal nodes of all tree topologies in $\mathcal{S}$.*

By Proposition 6, all tripartitions corresponding to internal nodes of all tree topologies in $\mathcal{S}$ pre-computed. Then, `OptimalTreeDP` uses a dynamic programming algorithm similar to the one formulated by Mirarab and Warnow 2015 to compute $\text{argmax}_{\hat{S}\in\mathcal{S}} W(\hat{S},\mathcal{G})$. □

PROPOSITION 5. *After each call to* `ColorLeafSet`$(\mathcal{L}^*,X,T,\mathcal{G},W)$ *with a $T\neq\emptyset$, $W[T] = \sum_{G\in\mathcal{G}} W(T,G)$.*

Proof. For a gene tree node w and a color X, let $\mathcal{L}_w^X$ denote the set of leaves in $\mathcal{L}_w$ colored by X. For an internal node w, let u,v be the children of w, p be the parent of w (if w is not the root), and e denote the branch (w,p). For a leaf i and internal node w, let $\mathcal{P}_{i,w}$ denote path between i and w and $s(\mathcal{P}) = 1-\prod_{\hat{e}\in\mathcal{P}}(1-s(\hat{e}))$. For leaves i,j, let $m(i,j)$ denote MRCA of i and j. Referring back to Table S1, we first establish the connection between recursive formulas of the algorithm and counter definitions.

- When $u_X = \sum_{i\in\mathcal{L}_u^X} e^{-l(\mathcal{P}_{i,w})}$, $v_X = \sum_{i\in\mathcal{L}_v^X} e^{-l(\mathcal{P}_{i,w})}$,
$$w_X := \Big((u_X+v_X)e^{-l(e)}\Big) = \sum_{i\in\mathcal{L}_w^X} e^{-l(\mathcal{P}_{i,w})}e^{-l(e)} = \sum_{i\in\mathcal{L}_w^X} e^{-l(\mathcal{P}_{i,p})}.$$
- When $u_{XX}^+ = \sum_{\{i,j\}\subseteq\mathcal{L}_u^X} e^{-l(\mathcal{P}_{i,j})}$, $v_{XX}^+ = \sum_{\{i,j\}\subseteq\mathcal{L}_v^X} e^{-l(\mathcal{P}_{i,j})}$,
$$w_{XX}^+ := u_{XX}^+ + v_{XX}^+ + u_X v_X = \sum_{\{i,j\}\subseteq\mathcal{L}_u^X} e^{-l(\mathcal{P}_{i,j})} + \sum_{\{i,j\}\subseteq\mathcal{L}_v^X} e^{-l(\mathcal{P}_{i,j})} + \sum_{i\in\mathcal{L}_u^X} e^{-l(\mathcal{P}_{i,w})} \sum_{j\in\mathcal{L}_v^X} e^{-l(\mathcal{P}_{j,w})}$$
$$= \sum_{\{i,j\}\subseteq\mathcal{L}_u^X} e^{-l(\mathcal{P}_{i,j})} + \sum_{\{i,j\}\subseteq\mathcal{L}_v^X} e^{-l(\mathcal{P}_{i,j})} + \sum_{i\in\mathcal{L}_u^X}\sum_{j\in\mathcal{L}_v^X} e^{-l(\mathcal{P}_{i,j})} = \sum_{\{i,j\}\subseteq\mathcal{L}_w^X} e^{-l(\mathcal{P}_{i,j})}.$$
- For $X\neq Y$, when $u_{XY}^+ = \sum_{(i,j)\in\mathcal{L}_u^X\times\mathcal{L}_u^Y} e^{-l(\mathcal{P}_{i,j})}$, $v_{XY}^+ = \sum_{(i,j)\in\mathcal{L}_v^X\times\mathcal{L}_v^Y} e^{-l(\mathcal{P}_{i,j})}$,
$$w_{XY}^+ := u_{XY}^+ + v_{XY}^+ + u_X v_Y + u_Y v_X$$
$$= \sum_{(i,j)\in\mathcal{L}_u^X\times\mathcal{L}_u^Y} e^{-l(\mathcal{P}_{i,j})} + \sum_{(i,j)\in\mathcal{L}_v^X\times\mathcal{L}_v^Y} e^{-l(\mathcal{P}_{i,j})} + \sum_{(i,j)\in\mathcal{L}_u^X\times\mathcal{L}_v^Y} e^{-l(\mathcal{P}_{i,j})} + \sum_{(i,j)\in\mathcal{L}_v^X\times\mathcal{L}_u^Y} e^{-l(\mathcal{P}_{i,j})}$$
$$= \sum_{\{i,j\}\subseteq\mathcal{L}_w^X\times\mathcal{L}_w^X} e^{-l(\mathcal{P}_{i,j})}.$$

- When $u^-_{XX}=\sum_{\{i,j\}\subseteq\mathcal{L}^X_u}e^{-l(\mathcal{P}_{i,j})}\prod_{\hat{e}\in\mathcal{P}_{m(i,j),w}}\big(1-s(\hat{e})\big)$, $v^-_{XX}=\sum_{\{i,j\}\subseteq\mathcal{L}^X_v}e^{-l(\mathcal{P}_{i,j})}\prod_{\hat{e}\in\mathcal{P}_{m(i,j),w}}\big(1-s(\hat{e})\big)$,

$$\begin{aligned}w^-_{XX}:=&(u^-_{XX}+v^-_{XX}+u_Xv_X)\big(1-s(e)\big)\\ =&\sum_{\{i,j\}\subseteq\mathcal{L}^X_u}e^{-l(\mathcal{P}_{i,j})}\prod_{\hat{e}\in\mathcal{P}_{m(i,j),p}}\big(1-s(\hat{e})\big)+\sum_{\{i,j\}\subseteq\mathcal{L}^X_v}e^{-l(\mathcal{P}_{i,j})}\prod_{\hat{e}\in\mathcal{P}_{m(i,j),p}}\big(1-s(\hat{e})\big)\\ &+\sum_{(i,j)\in\mathcal{L}^X_u\times\mathcal{L}^X_v}e^{-l(\mathcal{P}_{i,j})}\big(1-s(e)\big)=\sum_{\{i,j\}\subseteq\mathcal{L}^X_w}e^{-l(\mathcal{P}_{i,j})}\prod_{\hat{e}\in\mathcal{P}_{m(i,j),p}}\big(1-s(\hat{e})\big)\,.\end{aligned}$$

- When $u^-_{XY}=\sum_{(i,j)\in\mathcal{L}^X_u\times\mathcal{L}^Y_u}e^{-l(\mathcal{P}_{i,j})}\big(1-s(\mathcal{P}_{m(i,j),w})\big)$, $v^-_{XY}=\sum_{(i,j)\in\mathcal{L}^X_u\times\mathcal{L}^Y_u}e^{-l(\mathcal{P}_{i,j})}\big(1-s(\mathcal{P}_{m(i,j),w})\big)$, and $X\neq Y$, similarly,

$$w^-_{XY}:=(u^-_{XY}+v^-_{XY}+u_Xv_Y+u_Yv_X)\big(1-s(e)\big)=\sum_{(i,j)\in\mathcal{L}^X_w\times\mathcal{L}^Y_w}e^{-l(\mathcal{P}_{i,j})}\big(1-s(\mathcal{P}_{m(i,j),p})\big)\,.$$

- For $X\neq Y$, when $u_{XX|Y}=\sum_{\{i,j\}\subseteq\mathcal{L}^X_u}\sum_{k\in\left\{k'\in\mathcal{L}^Y_v:\mathcal{L}_{m(i,j)}\subsetneqq\mathcal{L}_{m(i,k')}\right\}}e^{-l(\mathcal{P}_{i,j})-l(\mathcal{P}_{k,w})}s\big(\mathcal{P}_{m(i,j),m(i,k)}\big)$, $v_{XX|Y}=\sum_{\{i,j\}\subseteq\mathcal{L}^X_v}\sum_{k\in\left\{k'\in\mathcal{L}^Y_v:\mathcal{L}_{m(i,j)}\subsetneqq\mathcal{L}_{m(i,k')}\right\}}e^{-l(\mathcal{P}_{i,j})-l(\mathcal{P}_{k,w})}s\big(\mathcal{P}_{m(i,j),m(i,k)}\big)$,

$$w_{XX|Y}:=\big(u_{XX|Y}+v_{XX|Y}+(u^+_{XX}-u^-_{XX})v_Y+u_Y(v^+_{XX}-v^-_{XX})\big)e^{-l(e)}\,.$$

Notice that $(u^+_{XX}-u^-_{XX})v_Y=\sum_{\{i,j\}\subseteq\mathcal{L}^X_u}\sum_{k\in\mathcal{L}^Y_v}e^{-l(\mathcal{P}_{i,j})-l(\mathcal{P}_{k,w})}s(\mathcal{P}_{m(i,j),w})$ and $u_Y(v^+_{XX}-v^-_{XX})=\sum_{\{i,j\}\subseteq\mathcal{L}^X_v}\sum_{k\in\mathcal{L}^Y_u}e^{-l(\mathcal{P}_{i,j})-l(\mathcal{P}_{k,w})}s(\mathcal{P}_{m(i,j),w})$. Thus,

$$\begin{aligned}w_{XX|Y}=&\sum_{\{i,j\}\subseteq\mathcal{L}^X_w}\sum_{k\in\left\{k'\in\mathcal{L}^Y_w:\mathcal{L}_{m(i,j)}\subsetneqq\mathcal{L}_{m(i,k')}\right\}}e^{-l(\mathcal{P}_{i,j})-l(\mathcal{P}_{k,w})}s\big(\mathcal{P}_{m(i,j),m(i,k)}\big)e^{-l(e)}\\ =&\sum_{\{i,j\}\subseteq\mathcal{L}^X_w}\sum_{k\in\left\{k'\in\mathcal{L}^Y_w:\mathcal{L}_{m(i,j)}\subsetneqq\mathcal{L}_{m(i,k')}\right\}}e^{-l(\mathcal{P}_{i,j})-l(\mathcal{P}_{k,p})}s\big(\mathcal{P}_{m(i,j),m(i,k)}\big)\,.\end{aligned}$$

- Similarly, when $u_{XY|Z}=\sum_{(i,j)\in\mathcal{L}^X_u\times\mathcal{L}^Y_u}\sum_{k\in\left\{k'\in\mathcal{L}^Z_u:\mathcal{L}_{m(i,j)}\subsetneqq\mathcal{L}_{m(i,k')}\right\}}e^{-l(\mathcal{P}_{i,j})-l(\mathcal{P}_{k,w})}s\big(\mathcal{P}_{m(i,j),m(i,k)}\big)$, $v^-_{XY|Z}=\sum_{(i,j)\in\mathcal{L}^X_v\times\mathcal{L}^Y_v}\sum_{k\in\left\{k'\in\mathcal{L}^Z_v:\mathcal{L}_{m(i,j)}\subsetneqq\mathcal{L}_{m(i,k')}\right\}}e^{-l(\mathcal{P}_{i,j})-l(\mathcal{P}_{k,w})}s\big(\mathcal{P}_{m(i,j),m(i,k)}\big)$, for distinct X,Y,Z,

$$w_{XY|Z}=\sum_{(i,j)\in\mathcal{L}^X_w\times\mathcal{L}^Y_w}\sum_{k\in\left\{k'\in\mathcal{L}^Z_w:\mathcal{L}_{m(i,j)}\subsetneqq\mathcal{L}_{m(i,k')}\right\}}e^{-l(\mathcal{P}_{i,j})-l(\mathcal{P}_{k,p})}s\big(\mathcal{P}_{m(i,j),m(i,k)}\big)\,.$$

- For distinct X,Y,Z,

$$\begin{aligned}w_{XX|YZ}:=&v_Xu_{YZ|X}+u_Xv_{YZ|X}+u_{XX|Z}v_Y+v_{XX|Z}u_Y+u_{XX|Y}v_Z+v_{XX|Y}u_Z\\ &+(u^+_{YZ}v^+_{XX}-u^-_{YZ}v^-_{XX})+(u^+_{XX}v^+_{YZ}-u^-_{XX}v^-_{YZ})\,.\end{aligned}$$

Notice that,

$$\begin{aligned}v_Xu_{YZ|X}=&\sum_{(h,i,j,k)\in\mathcal{L}^X_v\times\mathcal{L}^Y_u\times\mathcal{L}^Z_u\times\mathcal{L}^X_u}\delta_G(hk|ij)e^{-l(\mathcal{P}_{h,w})}e^{-l(\mathcal{P}_{i,j})-l(\mathcal{P}_{k,w})}s\big(\mathcal{P}_{m(i,j),m(i,k)}\big)\\ =&\sum_{(h,i,j,k)\in\mathcal{L}^X_v\times\mathcal{L}^Y_u\times\mathcal{L}^Z_u\times\mathcal{L}^X_u}\delta_G(hk|ij)e^{-l(\mathcal{P}_{i,j})-l(\mathcal{P}_{k,h})}s\big(\mathcal{P}_{m(i,j),m(i,k)}\big)\\ =&\sum_{(h,i,j,k)\in\mathcal{L}^X_v\times\mathcal{L}^Y_u\times\mathcal{L}^Z_u\times\mathcal{L}^X_u}w_G(hk|ij)\,.\end{aligned}$$

Similarly,

$$u_X v_{YZ|X} = \sum_{\substack{h\in\mathcal{L}_u^X\\ i\in\mathcal{L}_v^Y\\ j\in\mathcal{L}_v^Z\\ k\in\mathcal{L}_v^X}} w_G(hk|ij), u_{XX|Z}v_Y = \sum_{\substack{\{h,i\}\subseteq\mathcal{L}_u^X\\ j\in\mathcal{L}_u^Z\\ k\in\mathcal{L}_v^Y}} w_G(hi|jk), v_{XX|Z}u_Y = \sum_{\substack{\{h,i\}\subseteq\mathcal{L}_v^X\\ j\in\mathcal{L}_v^Z\\ k\in\mathcal{L}_u^Y}} w_G(hi|jk),$$

$$u_{XX|Y}v_Z = \sum_{\substack{\{h,i\}\subseteq\mathcal{L}_u^X\\ j\in\mathcal{L}_u^Y\\ k\in\mathcal{L}_v^Z}} w_G(hi|jk), v_{XX|Y}u_Z = \sum_{\substack{\{h,i\}\subseteq\mathcal{L}_v^X\\ j\in\mathcal{L}_v^Y\\ k\in\mathcal{L}_u^Z}} w_G(hi|jk).$$

Also,

$$\begin{aligned} u_{YZ}^+ v_{XX}^+ - u_{YZ}^- v_{XX}^- &= \sum_{(h,i)\in\mathcal{L}_u^Y\times\mathcal{L}_u^Z}\sum_{\{j,k\}\subseteq\mathcal{L}_v^X} e^{-l(\mathcal{P}_{h,i})-l(\mathcal{P}_{j,k})} \\ &- \sum_{(h,i)\in\mathcal{L}_u^Y\times\mathcal{L}_u^Z}\sum_{\{j,k\}\subseteq\mathcal{L}_v^X} e^{-l(\mathcal{P}_{h,i})-l(\mathcal{P}_{j,k})} \prod_{\hat{e}\in\mathcal{P}_{m(h,i),w}}(1-s(\hat{e})) \prod_{\hat{e}\in\mathcal{P}_{m(j,k),w}}(1-s(\hat{e})) \\ &= \sum_{(h,i)\in\mathcal{L}_u^Y\times\mathcal{L}_u^Z}\sum_{\{j,k\}\subseteq\mathcal{L}_v^X} e^{-l(\mathcal{P}_{h,i})-l(\mathcal{P}_{j,k})}\left(1-\prod_{\hat{e}\in\mathcal{P}_{m(h,i),m(j,k)}}(1-s(\hat{e}))\right) \\ &= \sum_{(h,i)\in\mathcal{L}_u^Y\times\mathcal{L}_u^Z}\sum_{\{j,k\}\subseteq\mathcal{L}_v^X} w_G(hi|jk). \end{aligned}$$

Similarly,

$$u_{XX}^+ v_{YZ}^+ - u_{XX}^- v_{YZ}^- = \sum_{\{h,i\}\subseteq\mathcal{L}_u^X}\sum_{(j,k)\in\mathcal{L}_v^Y\times\mathcal{L}_v^Z} w_G(hi|jk).$$

Notice that above cases count exactly once all quartets $hi|jk$ for all leaf nodes h,i colored X, j colored Y, k colored Z such that MRCA of h,i,j,k is w; namely,

$$w_{XX|YZ} = \sum_{\{h,i\}\subseteq\mathcal{L}_w^X}\sum_{j\in\mathcal{L}_w^Y}\sum_{k\in\{k':k'\in\mathcal{L}_w^Z,\mathrm{MRCA}(h,i,j,k')=w\}} w_G(hi|jk).$$

- We define $I(G)$ to be the set of internal nodes of gene tree G and $\mathcal{L}_G^X$ be the set of leaves of gene tree G with color X. It is trivial to verify that at the

$$Q = \sum_{G\in\mathcal{G}}\sum_{w\in I(G)} w_{AA|BC} + \sum_{G\in\mathcal{G}}\sum_{w\in I(G)} w_{BB|CA} + \sum_{G\in\mathcal{G}}\sum_{w\in I(G)} w_{CC|AB}.$$

At the end of procedure `UpdateCounters`, $\sum_{w\in I(G)} w_{XX|YZ} = \sum_{\{h,i\}\subseteq\mathcal{L}_G^X}\sum_{(j,k)\in\mathcal{L}_G^Y\times\mathcal{L}_G^Z} w_G(hi|jk)$. Thus, Q returned by `UpdateCounters` satisfies:

$$Q = \sum_{G\in\mathcal{G}}\left(\sum_{\substack{\{h,i\}\subseteq\mathcal{L}_G^A\\ (j,k)\in\mathcal{L}_G^B\times\mathcal{L}_G^C}} w_G(hi|jk) + \sum_{\substack{\{h,i\}\subseteq\mathcal{L}_G^B\\ (j,k)\in\mathcal{L}_G^C\times\mathcal{L}_G^A}} w_G(hi|jk) + \sum_{\substack{\{h,i\}\subseteq\mathcal{L}_G^C\\ (j,k)\in\mathcal{L}_G^A\times\mathcal{L}_G^B}} w_G(hi|jk)\right).$$

For tripartition $T=A|B|C$, note that by assumption, before the call, all the gene tree leaves are colored such that recoloring $\mathcal{L}^*$ by X would produce a coloring that matches T. Thus, at the end of the call to `ColorLeafSet`, for each gene tree G, we have $A\cap\mathcal{L}_G = \mathcal{L}_G^A$, $B\cap\mathcal{L}_G = \mathcal{L}_G^B$, and $C\cap\mathcal{L}_G = \mathcal{L}_G^C$. Then, the value returned by `UpdateCounters` satisfies:

$$Q = \sum_{G\in\mathcal{G}} W(A|B|C,G). \tag{20}$$

It can be easily verified that after each call to `ColorLeafSet`$(\mathcal{L}^*, X, T, \mathcal{G}, W)$, the species tree tripartition T matches the coloring of all gene trees as required by conditions of (20), concluding $W[T] = Q = \sum_{G\in\mathcal{G}} W(T,G)$. □

Proposition 6. *Before calling* `OptimalTreeDP` *in line 6 of Algorithm S1, lookup table W contains all tripartitions corresponding to internal nodes of all tree topologies in $\mathcal{S}$.*

Proof. Each $\hat{S} \in \mathcal{S}$ places i above a different node w of S creating a new node corresponding to tripartition $\mathcal{L}_w|\{i\}|\mathcal{L}_S - \mathcal{L}_w$ covered in line 24. Besides new nodes, each existing internal node w of S will correspond to a different tripartition after placing i onto S depending on the relative location of w and i. Let u,v denote the larger and the smaller child of w. Node w corresponds to $\mathcal{L}_u|\{i\}\cup\mathcal{L}_v|\mathcal{L}_S - \mathcal{L}_w$ if i is under u, corresponds to $\{i\}\cup\mathcal{L}_u|\mathcal{L}_v|\mathcal{L}_S - \mathcal{L}_w$ if i is under v, and corresponds to $\mathcal{L}_u|\mathcal{L}_v|\{i\}\cup\mathcal{L}_S - \mathcal{L}_w$ if i is above w. All three cases for each node w is covered in lines 20–22. □

Proof of Theorem 4

Theorem 4. *If there exists a species tree topology S^* satisfying that for each quartet subtree $ab|cd$,*

$$\sum_{G\in\mathcal{G}} w(ab|cd) > \max\left(\sum_{G\in\mathcal{G}} w(ac|bd), \sum_{G\in\mathcal{G}} w(ad|bc)\right), \tag{6}$$

then the output of Algorithm S2 will be S^*.

Proof. We start with a Corollary 1 of Theorem 3

Corollary 1. *Assuming (6), if S is compatible with the true tree S^*, then S' is compatible with S^*.*

By induction, W_i in line 8 of Algorithm S2 should contain all tripartitions of S^*, as at that time $S_i = S^*$ by Corollary 1. Consequentially, the output of Algorithm S2 must also be S^*. □

Proof of Proposition 4

Proposition 4. *The time complexity of Algorithm S2 is $O(kHn^2\log n)$.*

Proof. We begin with a proposition and a corollary.

Proposition 7. *Procedure* `ColorNode` *on any species tree node w takes $O(kH|\mathcal{L}_w|\log|\mathcal{L}_w|)$ time.*

Proof (sketch) of Proposition 7. We can prove this proposition by induction. For an internal node w with larger child u and smaller child v, if for some constant $C \geq \frac{6}{\log 2}$, `ColorNode` on u calls `UpdateCounters` at most $Ck|\mathcal{L}_u|(\log|\mathcal{L}_u|+1)$ times and `ColorNode` on u calls `UpdateCounters` at most $Ck|\mathcal{L}_v|(\log|\mathcal{L}_v|+1)$ times, then `ColorNode` on w calls `UpdateCounters` at most

$$\begin{aligned}
&Ck|\mathcal{L}_u|(\log|\mathcal{L}_u|+1) + Ck|\mathcal{L}_v|(\log|\mathcal{L}_v|+1) + 3k(|\mathcal{L}_v|+1)\\
\leq &Ck|\mathcal{L}_u|(\log|\mathcal{L}_w|+1) + Ck|\mathcal{L}_v|(\log\frac{|\mathcal{L}_w|}{2}+1) + 6k|\mathcal{L}_v|\\
\leq &Ck|\mathcal{L}_u|(\log|\mathcal{L}_w|+1) + Ck|\mathcal{L}_v|(\log|\mathcal{L}_w|+1) - Ck|\mathcal{L}_v|\log 2 + 6k|\mathcal{L}_v|\\
\leq &Ck|\mathcal{L}_w|(\log|\mathcal{L}_w|+1) + (6 - C\log 2)k|\mathcal{L}_v|\\
\leq &Ck|\mathcal{L}_w|(\log|\mathcal{L}_w|+1)\,\text{times.}
\end{aligned}$$

It is easy to verify that each `UpdateCounters` takes $O(H_G)$ time where H_G is the height of the gene tree, and thus `ColorNode` on node w takes $O(kH|\mathcal{L}_w|\log|\mathcal{L}_w|)$ time. □

COROLLARY 2 (Corollary of Proposition 7). *For any tree topology S with n species, the* `Place` *procedure on S takes $O(kHn\log n)$ time.*

`NaivePlacement` of taxon set T makes $r(|T|-3)$ calls to `Place`, each of which takes $O(kH|T|\log|T|)$ time. Thus, `NaivePlacement` takes $O(rkH|T|^2\log|T|)$ time and when $T=\mathcal{L}_S$ and $r=O(1)$, $O(rkH|T|^2\log|T|)=O(n^2kH\log n)$. □

Proofs of Theorems 6 and Theorem 5

THEOREM 6. *Under the conditions of Theorem 4, the DAC Algorithm S3 will output S^*.*

Proof. By Theorem 4, S_i in line 5 of Algorithm S3 are compatible with S^*. With Corollary 1, by induction, each S_e in line 21 of Algorithm S3 is compatible with S^*. Consequentially, W_i in line 26 contain all tripartitions of S^*, as at that time $S_i'=S^*$, and the output of Algorithm S3 must also be S^*. □

THEOREM 5. *When the inequality condition in Theorem 4 is satisfied, then the time complexity of the DAC algorithm is $O(n^{1.5+\epsilon}kH)$ with arbitrarily high probability.*

PROOF (SKETCH). From the inequality (6), we can trivially deduct that S^* is the species tree topology that maximizes the weighted quartet score, and each S_i in line 5 of Algorithm S3 is compatible to S^*. Also, each C_e in line 15 of Algorithm S3 equals the set of species under the edges coming off of the internal nodes on the path of S^* corresponding to e.

We now introduce a proposition

PROPOSITION 8. *With high probability,* $\max_{e\in E_{S_i}}|C_e|\le 2\sqrt{n}\log n+O(\sqrt{n})$.

Proof. For each pair of nodes u,v of S^*, let $C_{u,v}:=\{x:x\in\mathcal{L}_S, u \text{ is not on } \mathcal{P}_{x,v} \text{ and } v \text{ is not on } \mathcal{P}_{x,u}\}$. It is easy to verify that for every e of S_i, $C_e=C_{u,v}$ for some nodes u,v of S^*. For every u and v that are sufficiently apart so that $C_{u,v}$ has $2\sqrt{n}\log n+\omega(\sqrt{n})$ elements and a random T_i in line 4 of Algorithm S3,

$$P(C_{u,v}\cap T_i=\emptyset)=\left(1-\frac{1}{\sqrt{n}}\right)^{|C_{u,v}|}\le e^{-\frac{1}{\sqrt{n}}|C_{u,v}|}=\frac{1}{n^2}e^{-\omega(1)}=o\left(\frac{1}{n^2}\right).$$

By union bound, the probability that there exists a pair of nodes u,v of S^* such that $|C_{u,v}|\ge 2\sqrt{n}\log n+\omega(\sqrt{n})$ and $C_{u,v}\cap T_i=\emptyset$ is $o(1)$. Since, by definition, $C_e\cap T_i=\emptyset$ for every C_e, with high probability, there exists no C_e having $2\sqrt{n}\log n+\omega(\sqrt{n})$ elements. □

Since $|T_i|\sim\text{Binomial}(n,\frac{1}{\sqrt{n}})$, with high probability $|T_i|=O(\sqrt{n})$ and calling `NaivePlacement` on line 5 takes $O(n^{1.5}kH\log n)$ time. It is easy to confirm that $C_\emptyset=\emptyset$ and every call to `Place` takes as input a species tree topology of $O(\sqrt{n}\log n)$ species with high probability. Thus, with high probability, each call to `Place` takes $O(\sqrt{n}kH\log^2 n\log\log n)$ time and all $O(n)$ calls to `Place` takes $O(n^{1.5}kH\log^2 n\log\log n)$ time. Therefore, the time complexity of the DAC algorithm is $O(n^{1.5}kH\log^2 n\log\log n)=O(n^{1.5+\epsilon}kH)$ with high probability. □
